# Pancreatic cancer: molecular pathogenesis and emerging therapeutic strategies

**DOI:** 10.1038/s41392-025-02499-y

**Published:** 2026-01-03

**Authors:** Enrique Rozengurt, Guido Eibl

**Affiliations:** 1 Division of Digestive Diseases, Department of Medicine, David Geffen School of Medicine, University of California, Los Angeles, CA 90095 USA; 2 Department of Surgery, David Geffen School of Medicine, University of California, Los Angeles, CA 90095 USA

**Keywords:** Gastrointestinal cancer, Cell biology

## Abstract

Pancreatic ductal adenocarcinoma (PDAC) is an aggressive disease for which there is no effective treatment. A deep understanding of the mechanisms underlying the molecular pathogenesis, signaling pathways and risk factors leading to PDAC is of paramount importance for identifying novel targets, prognostic markers, preventive strategies, and signature markers for use in specific and personalized therapeutic procedures. Activating somatic mutations in the *KRAS* oncogene play a critical role in PDAC initiation and maintenance. Here, we highlight the complex interplay between KRAS signaling, the transcriptional coactivator YES1-associated protein (YAP) and Src family kinases (SFKs) in the pathogenesis of PDAC and drug sensitivity. We subsequently focused on diet-induced obesity, which has been correlated with an increased risk for developing PDAC in humans and mice and more severe clinical outcomes. Accumulating evidence also indicates that neural signals regulate critical functions of cancer cells, including their proliferation and dissemination, and that chronic stress promotes PDAC through the sympathetic nervous system via β-adrenergic receptors expressed by PDAC cells and other cells in the tumor microenvironment. Obesogenic mediators and stress neurotransmitters stimulate protein kinases, including PKA and PKD, which converge on CREB/ATF1 phosphorylation in PDAC cells. Since stress and obesity cooperate to promote the progression of PDAC, novel combinatorial strategies to prevent this devastating disease could be developed, repositioning FDA-approved drugs that are extensively used to treat cardiovascular and metabolic disorders and diseases. Finally, we review new advances in the treatment of PDAC, focusing on the discovery of novel drugs that directly inhibit KRAS and YAP function.

## Introduction

Pancreatic ductal adenocarcinoma (PDAC), which is the most prevalent type of pancreatic cancer, is a highly aggressive and lethal neoplastic disease. To date, surgical resection has remained the only curative therapy for PDAC, but 85% of tumors are diagnosed at an advanced stage^1^ and are therefore ineligible for surgery. Furthermore, in more than 80% of patients who undergo surgery, the resected tumors reappear within five years and, in some cases, as early as 6 months.^2^ The existence of micrometastases at the time of resection of the primary tumor drives subsequent recurrence.^3^ Additionally, the survival benefit of current neoadjuvant (delivered before surgery) and adjuvant (given after surgery) chemotherapeutic regimens is moderate, and relapses often take place.^4^ Consequently, the 5-year survival rate remains at a dismal 13%. Given the lack of success in treating existing PDAC, novel therapeutic and preventive approaches to counteract the progression of this devastating disease are urgently needed.^5^ A comprehensive understanding of the mechanisms underlying the molecular pathogenesis, signaling pathways and risk factors leading to PDAC is essential for identifying novel targets, prognostic markers, and preventive and interceptive strategies for use in specific and individualized treatments.

Accordingly, the first purpose of this study was to review the essential role of *KRAS-*activating mutations in the initiation of PDAC. Specifically, we highlight the interplay between KRAS signaling, the transcriptional coactivator YES1-associated protein (YAP) and Src family kinases (SFKs) in the pathogenesis of PDAC, including metabolic reprogramming and shaping of the tumor microenvironment. We also emphasize that *KRAS-*activating mutations are not sufficient to promote the evolution of preneoplastic lesions to overt PDAC. Consequently, our second purpose is to emphasize the importance of modifiable risk factors that function as tumor promoters of initiated pancreatic cells harboring *KRAS* mutations. In this context, we review the importance of diet-induced obesity, which is correlated with an increased risk for developing PDAC in humans and mice and more severe clinical outcomes of the disease. Accumulating evidence also indicates that neural signals regulate key functions of cancer cells, including their proliferation and dissemination, and that chronic stress promotes PDAC through the sympathetic nervous system via β-adrenergic receptors expressed by PDAC cells and other cells in the tumor microenvironment. Finally, we summarize recent advances in the development of new methodologies for the therapy, and prevention of PDAC, including the identification of new drugs that directly target KRAS and YAP.

## Epidemiology

The incidence of PDAC in the US is estimated to increase to 67,440 new cases in 2025.^6^ As indicated above, PDAC has the highest death rate of all major cancers estimated to reach 51,980 deaths in 2025,^6^ and it is currently the third leading cause of cancer mortality in men and women combined. Furthermore, epidemiological projections indicate that the mortality of patients with pancreatic cancer will continue to rise^7^ and exceed the number of deaths from colorectal cancer by 2030, making pancreatic cancer the second leading cause of cancer-related fatalities in the US and Europe by 2030. The incidence of pancreatic cancer is also increasing globally. A recent study revealed a greater relative increase in the incidence of PDAC among women younger than 55 years than among men,^8^ a finding subsequently validated in a large population.^9^ The remarkable rise in the incidence of pancreatic cancer is the result, at least in part, of a considerable increase in established risk factors, including diet-induced obesity, which will be discussed in subsequent sections.

## PDAC development: initiation and sequential progression

In most cases, PDACs develop through gradual progression from premalignant precursor lesions,^10,11^ the majority of which are pancreatic intraepithelial neoplasia (PanIN).^12^ PanINs are noncystic microscopic lesions that are usually diagnosed in histological preparations of tissue removed during surgery or in biopsy samples.^13^ PanINs advance from early, low-grade lesions (PanIN-1) to PanIN-3, also known as intraductal carcinoma or carcinoma in situ, and ultimately to overt PDAC. These lesions progressively accumulate key genetic alterations, which are also found in PDAC and are thus considered precancerous lesions.^12^ A major challenge in the field is to develop diagnostic modalities to detect advanced PanIN lesions before they progress to invasive PDAC.

In contrast to PanINs, which cannot be detected with current methods of imaging, macroscopic cystic pancreatic lesions are common in older patients and are benign in most cases. However, certain cystic precursor lesions, including intraductal papillary mucinous neoplasms (IPMNs) and mucinous cystic neoplasms (MCNs), also have the potential to advance to invasive PDAC.^14^ In contrast to the classical view of stepwise accumulation of somatic mutations, a subset of PDACs appear to evolve via extensive genomic rearrangements that often occur in a single catastrophic event (e.g., chromothripsis and chromoplexy)^15,16^ that results in rapid conversion to invasive PDAC.^17^

**Fig. 1:**
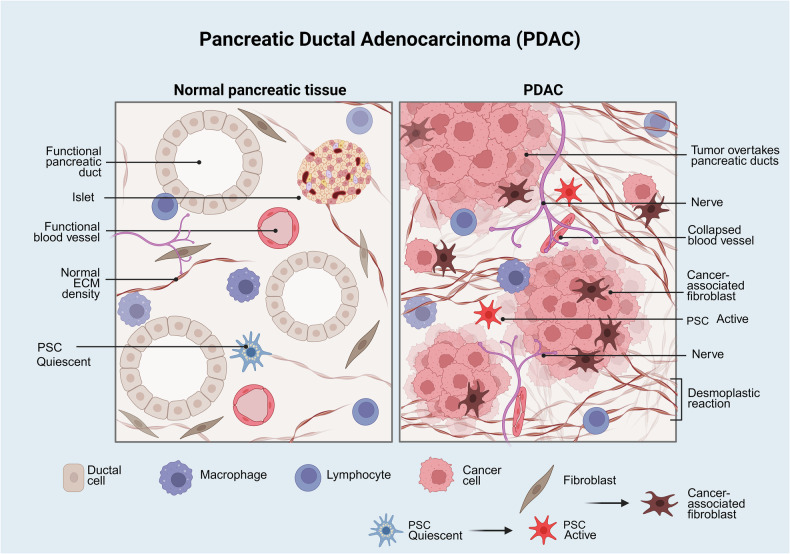
Scheme depicting the salient histological features of normal and PDAC. Unlike normal pancreatic cells, PDAC cells are surrounded by a microenvironment characterized by a profuse extracellular matrix, poor vascularization and bidirectional crosstalk between cancer cells and the cells residing in the pancreatic microenvironment. Pancreatic stellate cells (PCSs), which are in a quiescent state in normal tissue but interconvert into other cells during PDAC development, include cancer-associated fibroblasts. Other cells in the PDAC microenvironment include macrophages and other immune cells, endothelial cells and nerves of the autonomic system, which increase in size and number during PDAC development. Cancer-associated fibroblasts produce a dense collagen-rich extracellular matrix (desmoplastic reaction) that modulates tumor progression via paracrine signaling. Further details are provided in the text

A critical feature of PDAC is an acidic and hypoxic^18^ microenvironment characterized by a profuse extracellular matrix, poor vascularization^19^ and bidirectional transfer of signals between pancreatic cancer cells and the cells residing in the microenvironment.^20^ These cells include stellate pancreatic cells (SPCs), fibroblasts, macrophages and other immune cells, endothelial cells and nerves of the autonomic system,^21–23^ as shown schematically in Fig. 1. We discuss neural inputs in PDAC in a subsequent section of this review. In the normal pancreas, SPCs are in a quiescent state, but during PDAC development, SPCs interconvert into myofibroblasts, which are identified by the expression of α-smooth muscle actin (α-SMA). Pancreatic myofibroblasts play a critical role in creating a dense collagen-rich extracellular matrix (ECM), known as desmoplasia, that modulates tumor progression via paracrine signaling.^24^ Pancreatic cancer-associated fibroblasts (CAFs), derived from SPCs or other mesenchymal cells, are an increasingly heterogeneous population, and their secreted products exert tumor-promoting and tumor-suppressive effects.^25–28^ In turn, mutations in PDAC cells have a substantial influence on the stroma of PDAC.^29–31^ The extensive communication between different cells in tumors plays a decisive role in creating the highly immunosuppressive microenvironment that characterizes PDAC.^32,33^

### *KRAS* and its mutations in PDAC

Many studies have confirmed the vital importance of activating somatic mutations in the Kirsten rat sarcoma viral oncogene homolog (*KRAS*) oncogene in PDAC development.^34–36^ Multiple studies have shown that ~90% of PDACs harbor mutations in *KRAS*,^37,38^ a result corroborated by analyses of 3594 primary and metastatic PDAC samples from an international cohort.^39^ The detection of oncogenic *KRAS* mutations in more than 90% of low-grade PanIN lesions supports the notion that *KRAS* mutations constitute an early step in PDAC evolution.^36,40^ Consequently, the KRAS pathway is critically important in PDAC^41^ and is of major interest in this review.

RAS proteins function as small GTPases that fluctuate between an active state (with GTP-bound) and an inactive state (GDP-bound), which are distinguished by different conformations.^42^ The interconversion between these states is controlled by GTP exchange factors (RAS GEFs), which stimulate the GTP-bound state, and GTPase-activating proteins (RAS GAPs), which increase the rate of GTP hydrolysis, thereby reverting RAS-GTP to the GDP-bound state.^43^ In nongrowing cells, RAS is in a GDP-bound state and inactive. Cell stimulation via multiple receptor systems, including tyrosine kinases (RTKs), G protein-coupled receptors (GPCRs), integrins or cytokine cell-surface receptors, induces a fast and transitory increase in the concentration of RAS-GTP. Then, active RAS interacts with effector proteins at the plasma membrane that, in turn, regulate signaling pathways that initiate a proliferative response.

Approximately 91–98% of all missense *KRAS* mutations in PDAC occur at position G12,^44^ with a G12D single amino acid substitution being the most prominent (45%)^45^ and clinically aggressive.^46^ Other amino acid substitutions include valine (35%), arginine (17%), alanine, or cysteine.^47^ In a recent study involving 2433 patients, *KRAS* G12D and G12V mutations were associated with more severe PDAC *than* wild-type KRAS.^48^ Mutations at position G12 increase the level of the active state of the protein by reducing the rate of change of KRAS-GTP (active state) to KRAS-GDP (inactive state).^44^ In this manner, activating KRAS mutations induce persistent stimulation of downstream signaling cascades via direct interaction with the RAS-binding domain (RBD) of effector proteins.^49^ Downstream KRAS signaling must be finely tuned to promote cellular transformation since excessive activation might lead to growth arrest, for example, via the tumor suppressor RASSF1A,^50^ whereas little stimulation might not reach the signal intensity and duration necessary to stimulate pathways leading to cell proliferation.^51^

### Downstream of RAS

**Fig. 2:**
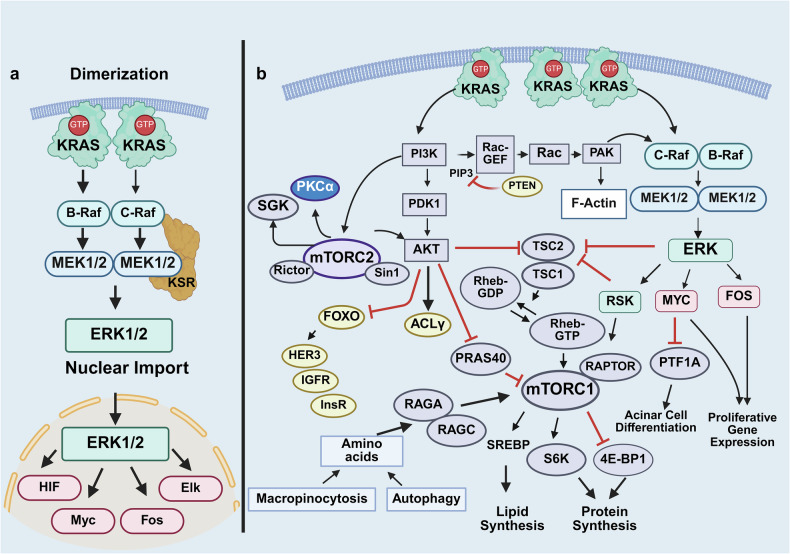
Active KRAS (KRAS-GTP) stimulates RAF/MEK/ERK and PI3K/AKT/mTOR complexes in the membrane and cytosol. **a** Dimers of KRAS-GTP bind the RBD (Ras binding domain) of RAF proteins, leading to their activation and thereby triggering the MEK/ERK cascade. Scaffold proteins, including KSR (Kinase Suppressor of RAS), increase the specificity and rate of the signaling module. KSR binds constitutively to MEK and binds to ERK in response to stimuli. Active ERK phosphorylates multiple substrates in the membrane, cytosol, and nucleus after undergoing nuclear import, including transcription factors implicated in cell proliferation. Pancreas transcription factor 1a (PTF1A) is a transcription factor critical for the maintenance of differentiated acinar cells. Repression of PTF1A by MYC facilitates acinar-ductal metaplasia. **b** In addition to stimulating the RAF/MEK/ER pathway, KRAS-GTP also binds to the RBD of p110α, a catalytic subunit of class I PI3K, leading to the synthesis of the second messenger PIP_3_ (phosphatidylinositol-3,4,5-trisphosphate), which in turn binds to PH domain-containing proteins, including PDK, AKT and mTORC2, and is downregulated by PTEN. PDK phosphorylates and activates AKT, which, in turn, phosphorylates and regulates multiple targets, including ATP citrate lyase (ACLY), a key enzyme in the mevalonate pathway (depicted in a subsequent figure), and the transcription factor FOXO1, which promotes the expression of tyrosine kinase receptors, including the insulin receptor (InsR) and the IGF receptor (IGFR). As described in the text, mTOR is the catalytic subunit of two different multiprotein complexes: mTORC1 and mTORC2. The regulation of mTORC1 by growth factors (via AKT, ERK, TSC1/TSC2 and Rheb) and amino acids is described in the text. mTORC2, downstream of PI3K, phosphorylates AKT, isoforms of protein kinase C (PKC α) and serum- and glucocorticoid-inducible kinase 1 (SGK). PIP3 also stimulates guanine exchange factors (GEFs) that activate the small G protein Rac, which in turn stimulates the serine/threonine kinases of the PAK family. References to the studies connecting the pathways as well as further details on the regulation of the network and its relevance to PDAC are in the text. Black lines: stimulatory connections. Red lines: inhibitory connections. Created in BioRender.com

The best characterized downstream pathways stimulated by active RAS are mitogen-activated protein (MAP) kinase (RAF/MEK/ERK/p90RSK) and phosphatidylinositol 3-kinase (PI3K)/AKT/mTOR, which promote cell multiplication, survival, and metastasis (scheme in Fig. 2a, b). The RAF/MEK/ERK pathway plays a vital role in PDAC development and is the central pathway downstream of mutant KRAS.^52^ Inactive RAF kinases reside in the cytosol in a monomeric autoinhibited state where the N-terminal domain impairs the activity of the catalytic domain.^53^ The binding of RAF kinases to KRAS dimers (Fig. 2a) via their RBD promotes RAF membrane localization, homo and heterodimerization and the release of autoinhibition, which ultimately stimulates RAF catalytic activation.^53,54^ There are three RAF isoforms in mammals (ARAF, BRAF and CRAF) and two related pseudokinases (KSR1 and KSR2), which function as scaffolds in the RAS/RAF/MEK/ERK pathway.^55^ In contrast to RAF and MEK kinases, which have narrow substrate specificity, ERKs phosphorylate multiple substrates,^56^ including the 90 kDa ribosomal S6 kinase (RSK), and both ERKs and RSK phosphorylate and inhibit tuberous sclerosis complex (TSC), as described below. A key step in the pathway is the translocation of ERK into the nucleus^,^^57^ where the proliferative program is promoted via ERK-mediated phosphorylation and activation of critical transcription factors, including ELK, FOS, and MYC.^58,59^ In turn, MYC binds and represses the transcriptional activity of pancreas-associated transcription factor 1a (PTF1a),^60^ thereby preventing pancreatic acinar cell differentiation.

The PI3K/AKT/mTOR signaling module,^61^ another pathway activated by RAS-GTP,^62^ plays a critical role in stimulating PDAC cell multiplication. Monomeric KRAS-GTP at the membrane interacts with the RAS-binding domain of the catalytic subunit of class I PI3K, thereby stimulating the synthesis of the second messenger PIP_3_ and downstream signaling (Fig. 2b). The tumor suppressor PTEN, a phosphatase that dephosphorylates PIP_3_, antagonizes the activation of the pathway. Accordingly, deletion of *Pten* induces the formation of precancerous cystic lesions in mice harboring active *Kras* in pancreatic cells.^63^ Downstream of the PI3K/AKT and ERK pathways, the serine threonine kinase (mTOR) operates as a catalytic subunit in two functionally different multisubunit complexes, mTORC1 and mTORC2 (Fig. 2b), which cooperate to promote the early stages of PDAC development.^64^ mTORC1, which is characterized by the Raptor subunit, phosphorylates and controls at least two regulators of protein synthesis, 40S ribosomal protein subunit S6 kinase (S6K) and the inhibitor of protein synthesis 4E-binding protein 1, referred to as 4EBP1. Rapamycin potently inhibits mTORC1, whereas mTORC2, characterized by Rictor and Sin1, resists acute inhibition by this agent. The tuberous sclerosis tumor suppressive complex, composed of TSC2 (tuberin) and TSC1 (hamartin), inhibits mTORC1 activation by stimulating the GTPase activity of the small G protein Rheb (ras homolog expressed in the brain), a potent activator of mTORC1 in its GTP-bound state,^65^ which is implicated in promoting PDAC development.^66^ Phosphorylation of TSC2 by AKT and/or ERK/RSK, as indicated in Fig. 2b, blocks the GTPase activity of TSC1/TSC2 toward Rheb, resulting in Rheb-GTP accumulation and mTORC1 activation. In addition, AKT phosphorylates proline-rich AKT substrate of 40 kDa (PRAS40),^67^ an inhibitory subunit of mTORC1, and RSK, a target of ERK, phosphorylates Raptor, thereby further facilitating mTORC1 activation. In turn, mTORC2, which is targeted by deleting Rictor in the pancreas, has emerged as an important pathway in PanIN formation and PDAC development.^68^ Importantly, amino acids regulate mTORC1 by modulating the GTP-bound states of Rag GTPases, which bind and recruit mTORC1 to the lysosomal surface,^69,70^ the site of Rheb-GTP activation. In this manner, mTORC1 activation requires the concerted stimulatory effects of both Rheb and Rags, clarifying the concomitant need for both growth factors and amino acids for mTORC1 activation.^71^ Downstream, mTORC1 participates in the regulation of protein and lipid synthesis.^72^

In addition to the RAF and PI3K cascades, RAS-GTP engages several additional effectors with RBDs,^49^ including RALA/B,^73^ which are implicated in the regulation of endocytosis, actin organization, proliferation and proinflammatory signaling through NFκB and RASSF1A, a tumor suppressor that stimulates proapoptotic signaling.^50^ It is plausible that different mutations of KRAS stabilize distinct conformational states that potentially differ in their affinity for the RBD of interacting effectors and thus alter the organization of the signaling network.^74^

The mechanisms by which KRAS proteins function on cell membranes to activate their effectors^43^ are of great interest and translational importance but remain incompletely understood. After sequential posttranslational modifications, RAS proteins localize to the cytoplasmic side of the plasma membrane bilayer. The concept that RAS dimerizes^75^ has attracted considerable attention. For example, using a tetracycline-regulated expression system and high-resolution microscopy, Nan et al.^76^ concluded that *KRAS*^*G12D*^ dimerizes as its concentration increases in cells. ERK activation was detected when the *KRAS*^*G12D*^ concentration reached a level that corresponded with dimerization.^76^ Subsequent reports support the notion that KRAS dimerizes though the precise mechanism of dimer formation via G protein domains or the C-terminus, and the role of dimerization in signal transduction remain important subjects of debate.^77–80^ Recently, a study using mass spectrometry demonstrated RAS dimerization on a lipid bilayer and defined the role of nucleotides, lipids, and palmitoylation in the regulation of oligomerization.^81^

Human PDAC cell lines harboring mutant KRAS do not exhibit high levels of constitutive MEK/ERK pathway activation when transferred to serum-free medium, a finding that has been extended to colon cancer cells with mutated RAS proteins.^82^ PDAC cells require additional stimulation via growth factor tyrosine kinase receptors or GPCRs to activate ERKs, thus revealing the requirement of extra inputs for stimulating KRAS signaling, possibly at the level of RAF.^82^ A recent study identified the FGF receptor (FGFR)2 as a key tyrosine kinase receptor that stimulates KRAS signaling in early-stage PDAC,^83^ and a similar role was attributed previously to EGFR.^84,85^

## Additional prevalent somatic mutations in PDAC: *CDKN2A*, *TP53*, and *SMAD4*

Although mutations in *KRAS* are initial steps in most cases of PDAC, they are not sufficient to promote invasive disease. Additional mutations (e.g., in tumor suppressor genes) or environmental stimuli, including obesity and chronic stress (developed in subsequent sections), are needed to induce high-grade PanIN lesions and PDAC. Accordingly, pancreatic cancers frequently involve inactivating alterations in tumor suppressor genes, including cyclin-dependent kinase inhibitor 2A (*CDKN2A*), encoding the CDK inhibitor p16^INK4A^, tumor protein p53 (*TP53*), and SMAD family member 4 (*SMAD4*), in 90%, 75%, and 50% of tumors, respectively.^86^ Consequently, alterations in cell cycle control, DNA damage sensing and TGF β-function are important oncogenic pathways in PDAC development that cooperate with KRAS signaling.^41,87^ Inactivation of *CDKN2A* is detected in PanIN-2 lesions, whereas mutations in *TP53* and *SMAD4* are found late in PDAC progression.^88^ *TP53* often undergoes missense mutation, resulting in gain of function rather than loss of expression.^89^

Although approximately 10% of pancreatic cancers lack *KRAS* mutations, RAS proteins are activated by receptor tyrosine kinases, including EGFR, which is also required in *Kras*^G12D^-driven PDAC.^39,84,85^ In a recent study, 44% of KRAS wild-type cases exhibited activating mutations in other components of the MAPK pathway, including activating *BRAF* mutations in approximately 25% of tumors.^90^ Overall, 3% of PDAC patients have mutations in *B-RAF*.^38,39^ Approximately 10% of pancreatic cancers have an inherited component linked to a variety of specific gene mutations or syndromes,^91^ including BRCA1/2 (associated with hereditary breast and ovarian cancer syndrome),^92,93^ Lynch syndrome,^94^ ATM^95^ and Peutz–Jeghers syndrome.^14,96^

The Wnt/β-catenin pathway is also implicated in the initiation and progression of pancreatic cancer.^97^ Hypoxia-inducible factor-2α (HIF-2α) is a transcription factor expressed early in pancreatic lesions that regulates WNT signaling by maintaining β-catenin levels during PanIN progression.^98^ Moreover, hypoxic conditions in pancreatic tumors stabilize HIF-2α, which cross talks with β-catenin, thereby promoting metabolic reprogramming and PDAC progression.^99^

## Genetically engineered mouse models of PDAC

Genetically engineered mouse models (GEMMs) of pancreatic cancer also support a key role of KRAS in PDAC development.^100,101^ Accordingly, the GEMM that best epitomizes the evolution of human PDAC includes the expression of a mutant *Kras* (*Kras*^*G12D*^) from the endogenous *Kras* locus through a Cre recombinase that is under the control of a pancreas-specific promoter, including *Ptf1a/p48* (*p48)* or *Pdx-1* [e.g., *LSL-Kras*^*G12D*^; *p48-Cre* mice, aka the KC model^102^]. This model closely replicates human disease as judged by morphological and genetic features, including the gradual evolution of PanIN lesions. The criteria for identifying murine PanIN-3 include papillary or micropapillary architecture, nuclear atypia, and the presence of actively dividing cells. Notably, *LSL-Kras*^*G12D*^*; p48-Cre* mice present fewer background tumors at a variety of sites than *LSL-Kras*^*G12D*^*; Pdx-1-Cre* mice do.^103^ The validity of the KC mouse model has been questioned on the basis that most cancers arise from somatic mutations that occur during adulthood.^104^ However, recent studies in various human cancers have changed this paradigm.^105^ Specifically, several driver mutations in hematological malignancies and solid tumors occur very early in life, sometimes during gestation, and clonal expansion occurs decades before cancer diagnosis.^105^

The expression of mutated *Kras* in pancreatic progenitor cells induces a process of cellular reprogramming known as acinar-to-ductal metaplasia (ADM), in which pancreatic acinar cells transdifferentiate into duct-like cells, leading to PanIN lesions followed by progression to PDAC.^106^ However, in the KC model, obvious PDAC generally occurs after a long latency (~9 months) and affects only ~10% of the animals.^100^ KC mice do not accrue additional alterations in tumor suppressor genes that are most commonly mutated in human PDAC (such as *Trp53* and *Smad4*) during PanIN progression.^101^ Furthermore, conditional mutations in *Cdkn2a*,^107^ *Trp53*^101^ *or Smad4*^108^ did not promote PanIN lesion or PDAC development. However, crossing KC mice with mice harboring *Trp53* deletions or mutations greatly accelerated PDAC development and shortened survival, as observed in *Kras*^*+/LSL-G12D*^; *Trp53*^*+/LSL-R172H*^; *Pdx1-Cre* (KPC mouse model) mice. External environmental factors, including diet-induced obesity, markedly accelerate PDAC progression in KC mice (developed in subsequent sections), phenotypically substituting for additional genetic mutations. Owing to slow PDAC development and responsiveness to environmental stimuli, the KC mouse model is useful for investigating risk factor-promoted PDAC development and preventive/interceptive strategies.

Most PDACs show allelic imbalances resulting in increased *Kras*^*G12D*^ gene dosage, which is associated with adverse prognosis^109^ and appears to require the loss of tumor suppressor genes, including *Cdkn2a* and *TP53*.^110^ Interestingly, gain of KRAS^G12D^ was linked to loss of wild-type *KRAS* in PDAC, suggesting that the wild-type allele of *Kras*^*G12D*^ functions as a tumor suppressor.^111,112^ The RAS dimerization model of signal transduction (discussed above) provides a possible mechanism that accounts for both the selective pressure that favors increased *Kras*^*G12D*^ gene dosage and elimination of the wild-type allele.^113^ A different mechanism explaining the tumor suppressive role of wild-type *Kras* involves functional activation of the transcription coactivator YAP^114^ in cells and tumors in which the nonmutated allele of *Kras* is lost.^114^ The Hippo/YAP pathway will be discussed in a subsequent section.

In addition to genetically engineered mouse models, the Oncopig model (somatic *LSL*-*KRAS*^G12D^-*TP53*^R167H^) has been used to create a porcine pancreatic cancer model to identify improved treatments and characterize the early pathogenesis of the disease, using an animal model more similar to human anatomy and physiology. A different approach of great interest is via the use of organoid models from normal and neoplastic murine and human pancreas tissues.^115,116^ Organoids better recapitulate the in vivo 3D architecture and interactions with other cells and thus mimic the architecture of the original tissue.^116^ Using single-cell RNA sequencing, a recent study revealed at least 15 different ductal cell populations in the normal murine pancreas with different organoid formation capacities and endocrine and exocrine differentiation potentials.^117^ Given the complexity of PDAC, advances in identifying novel therapeutic approaches for pancreatic cancer are likely to require the use of multiple in vitro and in vivo models of the disease.^118^

## PDAC subtypes and KRAS dependency

To identify subtypes of PDAC, investigators have carried out transcriptomic analyses using tumor samples and cell lines.^119–122^ These studies distinguished two clinically relevant subtypes, labeled basal-like (aka squamous or quasimesenchymal) and classical-pancreatic [reviewed previously^123,124^]. The classical and basal-like subtypes have been detected in multiple studies using samples from primary^119–121^ and metastatic tumors.^125,126^ Compared with the better differentiated classical subtype, the basal-like/squamous subtype is characterized by an undifferentiated phenotype, metabolic reprogramming through MYC activation, downregulation of the transcription factor GATA6 and worse survival.^127,128^ Several groups are using surrogate biomarkers and multiplex immunofluorescence to evaluate the utility of PDAC subtyping in the clinic.^127,129–131^ For example, low expression of GATA6 and high expression of Keratin 5 and the transcription factor TP63 are correlated with the basal-like subtype and resistance to chemotherapy.^129,130,132,133^ Similarly, the expression of high-mobility group A2 (HMGA2) protein complemented GATA6 in identifying the basal-like subtype of PDAC.^134^ Additionally, the two major subtypes of PDAC could be further subdivided into subgroups.^135,136^ The stromal, immune and neural compartments of the tumor microenvironment also play critical roles in promoting different PDAC subtypes.^137^ For example, the axon cue semaphorin 3A (SEMA3A), a member of the semaphorin family of secreted proteins, was found to enhance the malignant phenotype of the basal subtype of PDAC.^138^

Recent studies using single-cell and single-nucleus RNA-seq, as well as pancreatic organoids,^139^ have indicated that classical and basal-like/squamous PDAC cells are present in variable proportions in most PDACs (reviewed in ref. ^140^). Accordingly, recent studies have identified both basal-like and classical subtypes within individual PDAC tumors.^135,141,142^ PDAC subtypes are not linked to specific mutations but rather are related to the functions of distinct gene control networks and epigenetic events.^140^ Importantly, the proportion of the basal-like subtype of PDAC, as revealed by single-cell RNA sequencing, is a key determinant of chemotherapy response and patient outcome.^142,143^ Thus, the mechanisms leading to distinct PDAC subtypes are highly clinically important because converting the basal subtype to the classical subtype may provide an approach to increase the sensitivity of PDACs to therapeutic interventions.

To define the influence of the cell of origin on the subtype of PDAC, a recent study used a set of GEMMs with expression of oncogenic *Kras* and deletion of *Tp53* in either acinar or ductal cells.^144^ Specifically, the ductal cell–derived tumor signature is prominent in the basal-like/squamous subtype, whereas the acinar cell–derived tumor signature is pronounced in the classical subtype. In a separate study, single-cell suspensions of patient-derived organoids were introduced directly into the ducts of the murine pancreas.^145^ The subsequent lesions can be separated into two main subtypes: slow-growing with a glandular phenotype (like the classical subtype) and an aggressive and less glandular subtype with abundant desmoplasia (like the basal-like/squamous subtype). Taken together, these results support the notion that not only the cell of origin but also the contiguous microenvironment plays a significant role in determining the PDAC subtype.^144,145^

Although oncogenic *KRAS* plays a critical role in PDAC initiation, it is important to note that KRAS is dispensable for the viability of poorly differentiated PDAC cell lines.^146^ Accordingly, KRAS is not necessary for the survival of basal-like/squamous subtype tumors.^119,147,148^ For example, classical PDAC cell lines are more reliant on KRAS than are squamous PDAC lines, as demonstrated by the knockdown of KRAS expression.^119^ Accordingly, CRISPR/Cas-mediated *KRAS* knockout confirmed that the basal-like/squamous PDAC subtype is less dependent on mutated KRAS for survival and proliferation.^147^

To elucidate the role of oncogenic *Kras* in the initiation and maintenance of PDAC, additional GEMMs in which the expression of *Kras*^G12D^ can be turned on and off by the administration or removal of doxycycline were generated.^106,149^ Initial experiments examining individual mice indicated that turning off *Kras*^G12D^ expression resulted in tumor regression.^106,149^ Further analysis showed reversion of PDAC following the removal of doxycycline in many mice.^150^ While half of the recurrent PDAC re-expressed oncogenic *Kras*, the other half did not express this oncogene,^150^ indicating that another pathway promoted recurrence. Thus, the role of mutated KRAS in PDAC initiation can be separated from its influence on maintaining the survival of advanced PDAC.

As efforts to inhibit different KRAS mutants with specific drugs are becoming successful (discussed in a subsequent section), it is also increasingly appreciated that intrinsic or acquired resistance to these inhibitors is a major obstacle.^151^ Therefore, defining the molecular mechanisms that circumvent KRAS dependency is vital because they could identify novel targets for combination therapies for aggressive subtypes of PDAC. While the mechanisms involved in bypassing the need for RAS signaling are complex,^152,153^ increasing evidence indicates that activation of the transcriptional coactivators YAP/TAZ sidesteps the need for RAS signaling in a subset of PDAC cells. In what follows, we will focus next on the functions of these transcriptional regulators.

## Mechanisms that circumvent KRAS signaling in PDAC: the role of YAP/TAZ

The transcriptional coactivators Yes-Associated Protein (YAP) and its paralog WW-domain-containing Transcriptional co-Activator with PDZ-binding motif (TAZ) play critical roles in the control of fundamental programs for tumorigenesis, including proliferation, metabolic reprogramming, microenvironment signaling, ADM, development and patient survival.^154–159^ Here, we discuss how YAP/TAZ not only cooperate with KRAS in PDAC initiation but can also bypass KRAS signaling to maintain the viability of the basal subtype of PDAC.

### YAP regulation: succinct description

**Fig. 3:**
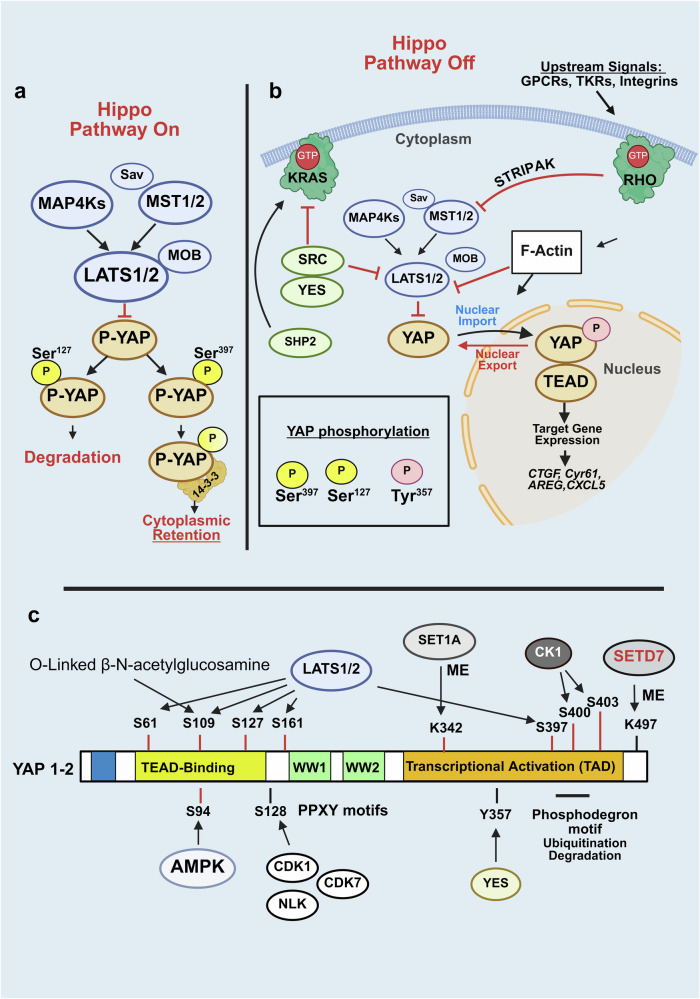
**a** Regulation of YAP localization and function through Hippo-dependent and Hippo-independent pathways. **a** When the tumor-suppressive Hippo pathway is active, the Mst1/2 kinases, in complex with Sav1, phosphorylate and activate Lats1/2 in complex with its regulatory protein MOB1/2. In addition to Mst1/2, MAP4Ks function as alternative kinases that phosphorylate Lats1/2. In turn, Lats1/2 phosphorylates YAP at highly conserved residues (e.g., Ser-127 and Ser-397). The phosphorylation of YAP at Ser127 promotes its binding to 14.3.3 proteins, leading to its cytoplasmic retention, whereas its phosphorylation at Ser397 induces degradation. **b** When the Hippo pathway is inactive, YAP is rapidly dephosphorylated, it moves into the nucleus, where it activates TEAD transcription factors (TEAD1–4) and stimulates the expression of multiple genes. The upstream signals that activate YAP include G protein-coupled receptor (GPCR) agonists, tyrosine kinase receptor (TRK) ligands and integrins. The activation of these receptors induces Rho activation (Rho-GTP), which promotes actin organization and striatin-interacting phosphatase and kinase (STRIPAK) activation, a multiprotein complex that represses the Hippo pathway by preventing Mst1/2 activation. The nonreceptor tyrosine kinase SRC regulates YAP localization via the phosphorylation and repression of LATS1/2, whereas YES1 directly phosphorylates YAP at Tyr357. SRC also phosphorylates and inhibits KRAS activity, thus eliciting opposite effects on the functions of YAP and KRAS. Notably, inhibitors that block both YES1 and SRC (e.g., dasatinib) prevent YAP nuclear accumulation but induce RAS/RAF/MEK/ERK activity by preventing KRAS tyrosine phosphorylation and inhibition. The tyrosine phosphatase and scaffold SHP2 activates RAS/ERK, at least in part by dephosphorylating KRAS, but inhibits YAP function by antagonizing its Tyr357 phosphorylation. Further details are provided in the text. **c** Schematic representation of the posttranslational modifications and subtypes of YAP1. The protein has either both WW domains (in YAP1–2 isoforms) or lacks the WW2 domain (in YAP1–1 isoforms). Each isoform can be divided into 4 subtypes generated by alternative splicing of the TAD, thus generating 8 possible forms of YAPs. YAP is modified by multisite phosphorylation, methylation and O-GlcNAcylation at specific residues by the indicated enzymes. Further details and references are provided in the text. Created in BioRender.com

A major repressor of YAP function is the tumor-suppressive Hippo pathway,^154,158^ which is represented by a serine/threonine kinase cascade in which the mammalian sterile 20-like kinase 1 or 2 (MST1 or MST2) binds and phosphorylates the scaffold protein Salvador homolog 1 (SAV1). The active MST–SAV1 complex then phosphorylates and activates the downstream kinases large tumor suppressor homologs 1 and 2 (LATS1 and LATS2) as well as the scaffold proteins MOB kinase activators 1A and 1B (MOB1A and MOB1B).^158^ Activated LATS1/2, in complex with its regulatory protein MOB1, phosphorylates YAP and its paralog TAZ, the major effectors of the Hippo pathway.^155,158^ There are other kinases and scaffolds in the Hippo pathway,^160–162^ including MAP4K1/2/3, MAP4K4/6/7 and NF2, which activate LATS1/2 and NDRs that phosphorylate YAP.^160,161^ In turn, angiomotins (AMOT, AMOTL1 and AMOTL2) stimulate LATS1/2 autophosphorylation and scaffold LATS1/2 to SAV1-MST1 and YAP.^162^ Similarly, WWC proteins function as scaffolds that promote MST-mediated phosphorylation and activation of LATS1/2.^163^ These studies demonstrated that Hippo is a complex system that regulates the nuclear/cytoplasmic distribution and stability of YAP/TAZ (Fig. 3a).

YAP and TAZ exhibit considerable homology and conserved consensus sequences phosphorylated by LATS1/2 (HXRXXS) but can also vary in their gene-regulatory functions.^164^ The phosphorylation of YAP by LATS1/2 at Ser127 and Ser397 (and equivalent residues in TAZ) induces cytoplasmic retention via complex formation with 14.3.3 proteins and promotes protein degradation, respectively. When YAP and TAZ are dephosphorylated at these residues, they translocate to the nucleus, where they bind and activate, chiefly, TEA-domain DNA-binding transcription factors (TEADs 1--4). Phosphorylation of YAP at Ser128 by Nemo-Like Kinase (NLK) interferes with LATS1/2-mediated phosphorylation of the adjacent Ser127, disrupts the interaction of YAP with 14.3.3 proteins and thereby stimulates its nuclear translocation.^165^ In turn, the YAP/TAZ–TEAD complex in the nucleus can cooperate with other DNA-binding partners.^166^ In this manner, nuclear YAP and TAZ promote the expression of multiple genes, including *CTGF* and *CYR61*, and display a level of functional redundancy^167,168^ but also differ in several ways. For example, overexpression of YAP downregulates TAZ expression, whereas increased TAZ expression does not reduce the level of YAP.^169^

Recent studies have identified other posttranscriptional modifications that control YAP localization and activity,^170^ including phosphorylation of serine/threonine residues by AMPK,^171,172^ CDK1,^173^ CDK7,^174^ MK5,^175^ MST4^176^ and NLK.^165^ The occurrence and impact of these and other modifications, including O-GlcNAcylation^177^ of YAP and TAZ in PDAC cells, remain largely unknown. Recently, another activating YAP modification, glutamylation, was identified in hypoxic PDAC.^178^ Phosphorylation of YAP at Tyr357 is another important posttranscriptional modification that regulates its nuclear/cytoplasmic shuttling, which will be discussed in a subsequent section. A summary of the YAP posttranslational modifications identified thus far is depicted schematically in Fig. 3b.

### YAP functions in PDAC development

YAP and TAZ are highly active in PDAC patient tumor samples, as indicated by their expression and/or localization.^150^ Furthermore, recent reports identified YAP expression as an independent prognostic marker for overall survival in patients with PDAC.^148,179^ In GEMMs, *Yap* deletion from pancreatic epithelial cells in mice does not hamper normal pancreatic development or homeostasis.^180,181^ In contrast, *Yap* is necessary for the formation of PanIN lesions and progression to PDAC in KC or KPC mice.^180^ Furthermore, deletion of both *Yap* and *Taz* blocked acinar-ductal metaplasia after cerulein-induced pancreatitis in KC mice,^181^ at least in part, by impairing CTGF upregulation.^182^ Yap stimulates *Myc* expression and collaborates with this oncogenic transcription factor to reprogram metabolism.^183^ YAP also promotes a microenvironment in the pancreas that suppresses the immune system through myeloid-derived suppressor cells.^184^ Although Yap and Taz cooperate with Kras in the initial stages of PanIN formation and PDAC development, substantial evidence also indicates that YAP function can sidestep the need for oncogenic KRAS for advanced PDAC maintenance.^102,150,185–187^ In agreement with the notion that YAP can substitute for KRAS, YAP is highly active in PDAC of the basal-like/squamous subtype.^148^ Furthermore, WNT5A, a ligand that induces YAP activation via noncanonical WNT signaling^,^^188^ is overexpressed in PDAC^148^ and replaces oncogenic *Kras* in tumor maintenance.^148^ The overexpression of the active YAPSer127A mutant in PDAC cells of the classical subtype enhanced their malignant phenotypes and transformed them into the squamous subtype.^148^ These studies lend further support to the notion that YAP signaling is important for promoting the basal-like/squamous PDAC subtype.

In conclusion, increasing evidence indicates that YAP is a major tumor-promoting transcription factor in PDAC and plays a significant role in circumventing KRAS function in the basal-like/squamous subtype of the disease. Additional findings showing that YAP interacts with other important factors in PDAC tumorigenesis, including ZEB1,^189,190^ the chromatin-remodeling complex SWI/SNF,^36,191^ AP1^192,193^ and p53 family members, including p63 isoforms,^194^ further substantiate the concept that YAP/TAZ play critical roles in PDAC development.

### Regulation of YAP and RAS by tyrosine phosphorylation mediated by SRC family kinases

Although YAP is essential for PDAC development, mutations in genes encoding Hippo pathway components are virtually nonexistent, underscoring the importance of defining upstream pathways that regulate the nuclear/cytoplasmic distribution and activity of YAP/TAZ. These include signals induced via receptor-mediated pathways, integrins and mechanical cues from the microenvironment. Our own work demonstrated that crosstalk between the insulin/IGF-1 receptor and GPCR systems^195,196^ induces robust nuclear YAP localization, decreases phosphorylation at sites targeted by LATS1/2 and stimulates transcriptional coactivator activity.^197^ Alterations in actin cytoskeletal organization downstream of Rho modulate YAP/TAZ activation in many cell types.^154,166^ In addition, Rho regulates YAP/TAZ activity through stimulation of the striatin-interacting phosphatase and kinase (STRIPAK) complex, which dephosphorylates and inactivates the upstream kinases of the Hippo pathway^198^ (Fig. 3a).

The nonreceptor Src family of tyrosine kinases (SFKs) regulate key cellular processes in cancer cells, including cytoskeletal organization, migration, proliferation, invasion and metastasis, in multiple solid tumors,^199,200^ including PDAC.^201,202^ SFK comprises 12 members (SRC, FYN, YES1, YRK, LYN, HCK, FGR, BLK, LCK, BRK, SRM, and FRK), three of which, SRC, FYN, and YES1, are expressed prominently in human pancreatic cancer cell lines.^203^ YES1 is markedly upregulated in PDAC, and increased expression of YES1 is significantly associated with unfavorable prognosis (survival) in PDAC^204^ and other cancer types.^205^ In addition, YES1 mRNA expression is closely correlated with YAP expression in PDAC. Preclinical studies have demonstrated that SFK activity is increased during progression to invasive PDAC^206^ and that SFK cooperates with Kras to promote PDAC development.^207^

SFKs regulate YAP function through multiple mechanisms, including Hippo-dependent and Hippo-independent pathways.^208–212^ Hippo-dependent control of YAP by SFK includes direct phosphorylation of LATS1 at multiple tyrosine residues, which inhibits its catalytic activity.^208^ Alternatively, SFK may regulate YAP activity via Hippo-independent pathways, including direct phosphorylation of YAP at Tyr357.^204,210^ Stimulation of human PDAC cells with the GPCR agonist neurotensin and insulin induces an SFK-dependent increase in YAP phosphorylation at Tyr357. The use of YAP mutants and a selective inhibitor of exportin 1 (XPO-1), the major nuclear‒cytoplasmic exportin, indicated that YAP phosphorylation at Tyr357 decreases its nuclear export, thereby leading to the accumulation^204^ of YAP in the nucleus. Importantly, the expression of YAP, importin 7 (IPO7), a regulator of YAP nuclear import, and YES1, a tyrosine kinase of the SRC family that phosphorylates YAP (see above), are unfavorable prognostic markers in PDAC.

Taken together, these findings suggest that SFK inhibitors could be useful in PDAC therapy. An early study revealed that the administration of the potent SFK inhibitor dasatinib^213^ prevented metastatic dissemination in preclinical models of PDAC but did not hinder the growth of the primary tumor.^206^ Disappointingly, clinical trials in PDAC patients using dasatinib in combination with gemcitabine^214^ have not shown significant clinical benefits. The identification of opposite effects of SFK on YAP and KRAS, as discussed below, provides a plausible mechanistic explanation for the disappointing results of these clinical trials.

Several studies have demonstrated that SRC phosphorylates wild-type and oncogenic (G12D) KRAS at tyrosine residues (Tyr32 and Tyr64), thereby inhibiting KRAS function, including RAF/MEK/ERK pathway activation.^215–217^ In contrast, the pro-oncogenic tyrosine phosphatase SHP2,^218,219^ which dephosphorylates KRAS,^220^ promoted RAF/MEK/ERK signaling. Consequently, tyrosine phosphorylation of KRAS in PDAC could interfere with tumor development via KRAS inactivation.^215,220^ In this context, SFK inhibitors can induce RAS hyperactivation, thereby leading to enhanced RAF/MEK/ERK signaling in PDAC cells. In line with this notion, the exposure of PDAC cells to dasatinib increased ERK activity, which was prevented by exposure to the MEK inhibitor trametinib.^204^ Interestingly, PDAC cell treatment with both dasatinib and trametinib blocked YAP phosphorylation at Tyr357 and ERK activation and abolished cell multiplication. Moreover, the combination of these inhibitors prevents the growth of PDAC cell xenografts.^204^ These findings revealed a complex interplay between KAS, YAP and SFK, as schematically represented in Fig. 3. These results also provide a plausible explanation for the disappointing results obtained with dasatinib in clinical trials and exemplify the difficulty of targeting signal transducers unless a detailed mechanistic understanding of the signaling network is available.

## Metabolic reprogramming in PDAC

The oncogenic mutations in *KRAS* and other genes in PDAC cells, as well as the acidic and hypoxic microenvironment surrounding these cells, induce striking adaptive changes in the metabolic pathways of PDAC cells.^221–223^ A central alteration is a marked increase in the rate of glycolysis even in the presence of oxygen (aerobic glycolysis), driven by increased glucose uptake and increased activity of the rate-limiting enzymes of the glycolytic pathway. *SLC2A1*, encoding the glucose transporter GLUT1, is prominently expressed in PDAC as well as in other cancer types and is associated with chemoresistance.^224^ PDAC also expresses another glucose import system, the Na^+^-dependent glucose transporter (SGLT), which has also been implicated in PDAC cell survival.^225^

Glucose transferred from the extracellular medium to inside PDAC cells across the plasma membrane via glucose transporters is phosphorylated by hexokinases (HKs) to glucose-6 phosphate, thus channeling it into the glycolytic pathway. Tumor cells, including PDAC cells, preferentially express HK2, an isoform of the HK family that localizes to the outer membrane of mitochondria and other sites in the cell.^226^ Furthermore, PDAC, like other gastrointestinal cancers, depends on increased activity of rate-limiting enzymes in the glycolytic pathway,^227^ including phosphofructokinase isoenzyme 1 (PFK1) and pyruvate kinase isoenzyme 2 (PKM2), which are expressed in many tumor cells, including those in PDAC. PFK2 (6-phosphofructo-2-kinase/fructose-2,6-bisphosphatase) generates fructose-2,6-bisphosphate, a potent allosteric activator of PFK1. In turn, fructose-1,6-bisphosphate, produced by PFK1, is a powerful allosteric activator of PKM2, thereby increasing glycolytic flux. The prominent increase in the glycolytic pathway in PDAC was documented in vivo^149^ and further confirmed in a recent analysis of gene datasets.^228^ In addition to their metabolic functions, these glycolytic enzymes also play a nonmetabolic role in the regulation of gene expression.^229^

Lactate, a byproduct of the glycolytic pathway produced by lactate dehydrogenases, has long been considered a waste product of glycolysis. Lactate is increasingly recognized as a signaling molecule that participates in the regulation of gene expression via the process of lactylation,^230–232^ the addition of a lactyl group to lysine residues in histones^230^ and other proteins.^231^ Lactylation has attracted great interest as a novel mechanism of metabolic reprogramming^231,233^ and reshaping of the PDAC microenvironment toward immunosuppression.^234,235^ In line with enhanced aerobic glycolysis and lactate production, histone lactylation is elevated in PDAC and is associated with an unfavorable prognosis.^236^

Glutamine has emerged as a critical amino acid for cancer cells with mutated KRAS.^237^ Although glutamine is a nonessential amino acid, PDAC cells depend on exogenous glutamine metabolism for their anabolic processes, including serving as a carbon source for lipid biosynthesis and a nitrogen source for nonessential amino acid and nucleotide biosynthesis. Importantly, glutamine is metabolized through a noncanonical pathway in PDAC cells^238^ involving the conversion of glutamine-derived aspartate to oxaloacetate, malate and pyruvate. These reactions increase NADPH levels and help maintain the cellular redox state in the cell.^227^ HIF-2α, a critical transcription factor in hypoxic PDAC, promotes noncanonical glutamine metabolism via activation of the PI3K/mTORC2 pathway.^239^ In addition, glutamine is also utilized by PDAC to shape the microenvironment and immunosuppression^240^ and for the generation of ornithine, leading to the synthesis of polyamines.^241^ Glutamine addiction is a metabolic vulnerability of PDAC cells that can be targeted with glutamine antagonists.^242^ Similarly, the dependence of PDAC on lipid metabolism is a vulnerability that can be targeted in PDAC.^243^

PDAC also involves increased scavenging mechanisms, including autophagy and micropinocytosis, a non-selective uptake of extracellular fluid, to supply glutamine and other amino acids, thereby maintaining tumor survival in a stressful microenvironment.^244^ Autophagy is also needed to maintain the intracellular iron pool required for mitochondrial function and PDAC cell growth.^245^ The role of autophagy in PanIN formation and PDAC development is complex: initially, autophagy prevents early PanIN development, but subsequently, this process is necessary for metabolic adaptation in established PDAC.^246^

As mentioned in a previous section, YAP plays a critical role in promoting PDAC, at least in part, via metabolic reprogramming. YAP proteins enhance glucose metabolism by increasing the levels of the glucose transporters GLUT1 and GLUT3 and the glycolytic flux by increasing the expression of rate-limiting glycolytic enzymes. Importantly, YAP also increases amino acid uptake by promoting the expression of the amino acid transporters SLC1A5, SLC7A5, SLC38A1 and SLC1A3 and enzymes related to glutamine metabolism.^247,248^ In this manner, YAP increases the availability of amino acids, thereby stimulating mTORC1 activation, as indicated in the preceding section.

## Modifiable environmental risk factors that promote PDAC development: obesity and neural inputs

Although activating mutations in oncogenes and inactivation of tumor suppressor genes play critical roles in the initiation of cancers, including PDAC, the crucial role of the promotion of initiated cells in the pathogenesis of this disease is increasingly recognized.^249^ As mentioned previously, a number of driver mutations in hematological malignancies and solid tumors occur very early in life, sometimes during gestation, and clonal expansion occurs decades before cancer diagnosis.^105^ Recent studies in other cancer types support the notion that normal cells harbor oncogene mutations that only progress to overt cancer in response to modifiable environmental factors.^250,251^ These recent studies, which exploit new technologies, reached conclusions that are reminiscent of early studies^249^ that separated carcinogenesis into initiation and promotion stages and reinforce the notion that avoidance of promotion is an important approach in cancer prevention.^252^ Consequently, we focus on two major risk factors for PDAC, obesity and chronic stress, which stimulate the promotion of oncogene-initiated pancreatic cells into overt PDAC in preclinical models and are important risk factors for human PDAC.

### Diet-induced obesity

In addition to risk factors such as smoking and chronic pancreatitis, diet-related metabolic disorders, including obesity, type 2 diabetes mellitus (T2DM) and metabolic syndrome, are associated with an increased risk for developing PDAC and other cancers.^34,253–261^ Compared with total adiposity, abdominal adiposity is a more important risk factor for PDAC.^258^ Persistent metabolic syndrome, characterized by elevated waist circumference, fasting hyperglycemia, high triglyceride levels and hypertension, is associated with a 30% increase in the risk of PDAC.^262^ In addition to risk, increased PDAC-related mortality was also observed in overweight and obese individuals compared with controls.^263^ We and others have replicated these connections in preclinical mouse models of PDAC.^264–266^ Specifically, diet-induced obesity (DIO) markedly accelerates the transition of low-grade PanINs to high-grade PanINs and overt PDAC in KC mice^34,264^ via multiple mechanisms, including cell proliferation and sustained inflammation.^34^ Accordingly, the administration of the antidiabetic drug metformin^267,268^ or increased physical activity^269,270^ markedly diminished obesity and PDAC development induced by DIO in this model.

Diet-related metabolic disorders are multilayered, but common features include peripheral insulin resistance, compensatory overproduction of insulin, and increased bioavailability of insulin-like growth factor-1 (IGF-1). Insulin and IGF-1 act as potent growth factors for PDAC cells and consequently are implicated in the promotion of PDAC and other cancers.^271^ Given the arrangement of the pancreatic microcirculation, local insulin released by β cells could act directly on insulin receptors expressed on the surface of exocrine pancreatic cells. Insulin and IGF1 stimulate glucose uptake, activate the PI3K/AKT/mTOR pathway,^272^ potentiate crosstalk with GPCR agonists^196^ and increase YAP function in human PDAC cells.^197^ Accordingly, a recent study demonstrated that insulin receptors expressed in the pancreatic acinar cells of KC mice subjected to DIO play critical roles in mediating hyperinsulinemia-driven PanIN development.^273^

Other prominent hormonal mediators in obesity-induced PDAC include leptin, an appetite-suppressing hormone released by adipocytes. An increase in the circulating leptin concentration has been observed in PDAC patients^274^ and is associated with increased PDAC risk in men.^275^ Interestingly, a recent study indicated that YAP/TAZ–TEAD stimulates leptin expression by directly interacting with an upstream enhancer site of the leptin gene.^276^ Bioactive peptides produced by neuronal and gastrointestinal (GI) cells (the brain‒gut axis) are also implicated in obesity-associated PDAC development. These peptides include neurotensin,^277–279^ glucagon-like peptide-1 (GLP-1),^280^ neuropeptide Y (NPY), peptide YY, gastric inhibitory peptide (GIP) and cholecystokinin.^281^ A positive correlation between obesity and PDAC risk is well established, but the mechanism(s) involved remain incompletely understood. Consistent with the concept that obesity influences the promotion of PDAC, diet-induced obesity does not produce additional driver mutations in mice or humans.^282^ An alternative notion is that GI peptides released from enteroendocrine cells in the gut or β cells of the pancreas play important endocrine or paracrine roles in stimulating the proliferation of pancreatic cells harboring an initiating *KRAS* mutation, acting synergistically with insulin.^34,273,283^ Accordingly, we demonstrated that the neuropeptide and gastrointestinal hormone neurotensin induces rapid signaling events^195,284^ and acts as a potent mitogen for human PDAC cells,^195,197^ which is in line with the notion that these obesogenic mediators play a significant role in PDAC promotion of initiated cells. This hypothesis also provides an explanation for most epidemiological studies that clearly point to obesity as a risk factor for (early) PDAC development. Mechanistically, obesity-related mediators reinforce KRAS activation^266^ and stimulate downstream pathways, including the ERK pathway.^34^ DIO also increased the protein levels of YAP and TAZ in the pancreas of KC mice, which were decreased by the administration of metformin. Although the mechanisms by which obesity promotes PDAC are complex, obesity mediators stimulate the function of two key molecular mediators of PDAC, namely, KRAS and YAP.

In what follows, we will concentrate on an emerging new area of research highly relevant to the promotion of PDAC and other cancers, namely, the impact of neural inputs and chronic stress on cancer development and the interplay between chronic stress and obesity in promoting advanced PanIN lesions and PDAC.

### Neural inputs in PDAC

Mounting evidence supports the notion that neural signals induce and regulate critical functional capabilities of cancer cells, known as the hallmarks of cancer,^285^ including cellular proliferation, resistance to cell death, invasion and metastasis, and tumor-promoting inflammation.^285,286^ Nerves in the pancreatic tumor microenvironment are known to be surrounded and invaded by cancer cells.^287,288^ There is increasing recognition that perineural invasion (PNI) is an important negative prognostic factor in PDAC and is associated with the characteristic presence of intractable pain in PDAC patients.^287–290^ While initially considered inert bystanders, recent studies have demonstrated a critical role of neural inputs in modulating cancer initiation and progression.

Various preclinical models of a variety of cancers have shown that nervous system activity can regulate cancer initiation, progression, and metastasis^286^ and immune cell function in the TME.^291^ Experimental denervation impaired tumor formation and progression in animal models of prostate,^292^ gastric,^293^ and pancreatic^294,295^ cancer. Moreover, bidirectional communication between neurons and cancer cells accelerates tumor development and progression.^295–297^ Specifically, cancer cells induce the outgrowth of nerves in the tumor microenvironment through paracrine-acting neurotrophic factors (e.g., NGF, BDNF) and axon guidance molecules, and in turn, nerves release neurotransmitters in the tumor microenvironment that stimulate cancer cell proliferation, invasion, and metastasis. Stromal cells residing in the tumor microenvironment also contribute to neuronal remodeling via paracrine cytokines, including leukemia inhibitory factor (LIF)^,^^298^ and neural progenitors from the brain infiltrate prostate cancer in mouse models of this disease.^299^ In addition, pancreatic nerves impact cancer cell metabolism by releasing serine, which sustains the growth of serine-dependent PDAC cells when the supply of this amino acid is restricted.^300^ Although perineural invasion is recognized as the most common mode of local invasion in PDAC, the active tumor-promoting role of nerves in the development of cancer in the pancreas, a highly innervated organ, is a novel key feature in the pathogenesis of this devastating disease.

In line with the notion that neural signals are implicated in PDAC development, the pancreas is innervated by fibers of the autonomic nervous system^301^ that increase in size (neural hypertrophy) and number (increased neural density) early in the course of pancreatic cancer.^288,302–304^ Pancreatic nerves are increasingly recognized as important components of the pancreatic tumor microenvironment that cross-talk with transformed epithelial and stromal cells, including fibroblasts and endothelial and immune cells^,^^285,305–308^ and are likely to regulate tumor growth differently depending on the quantity and type of innervation.^309^ In turn, PanIN cells promote the growth of sympathetic axons via the release of Netrin-1.^310^ β-adrenergic GPCRs are endogenously expressed by pancreatic tumor cells,^311^ and in vitro studies suggest that tumor cell behavior, including proliferation, is sensitive to β-adrenergic signaling.^312,313^ The cells in the PDAC microenvironment, comprising immune and endothelial cells, also express these receptors. These findings indicate a critical role of neural inputs and remodeling in the initiation and promotion of PDAC and identify a new point of intersection between neuroscience and cancer biology.^314^

### Chronic stress and PDAC

**Fig. 4:**
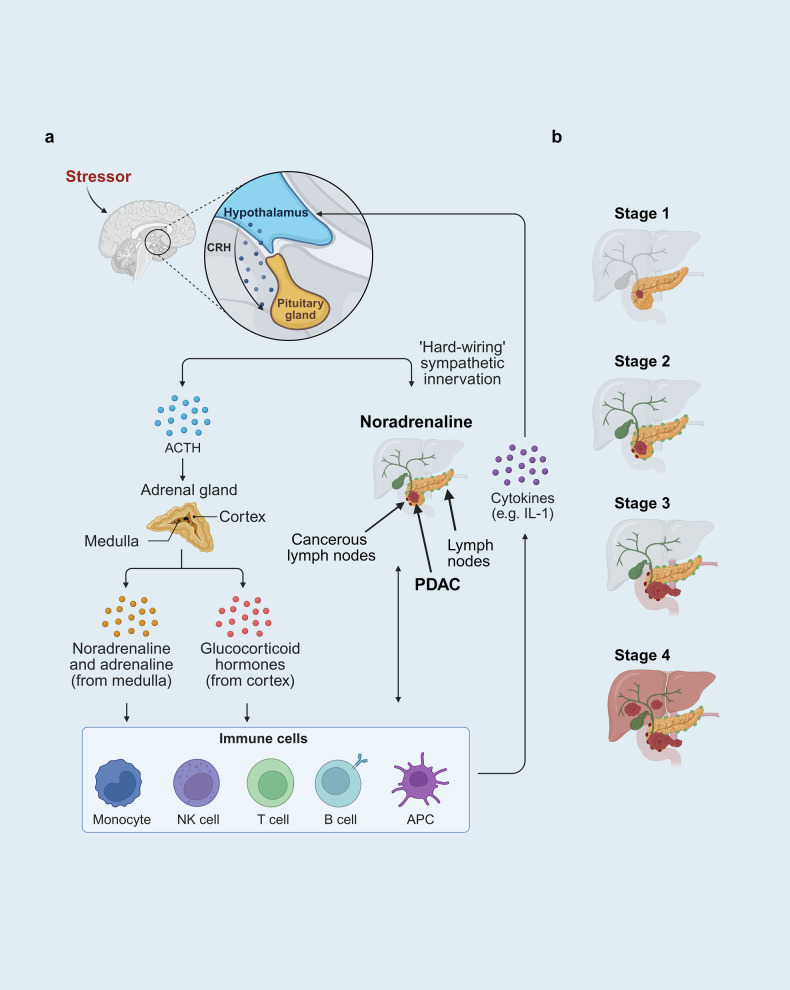
Chronic stress accelerates PDAC development. **a** The mechanisms linking chronic stress and pancreatic cancer development include neuroendocrine alterations in the hypothalamus‒pituitary‒adrenal (HPA) axis controlling corticotropin‒releasing hormone (CRH)-induced adrenocorticotropic hormone (ACTH) release and glucocorticoid output from the adrenal gland and the sympathetic nervous system (SNS), which regulate catecholamine levels. The SNS, which innervates virtually every major organ system, including the pancreas, responds to stress perceived by the brain by releasing noradrenaline from sympathetic nerve endings and adrenaline from the adrenal medulla into the circulation. Pancreatic cells and immune cells express β-adrenergic receptors that initiate cAMP signaling, leading to PKA activation, as explained in the text. The SNS-stimulated cAMP/PKA axis is implicated in immune evasion and pancreatic cancer cell proliferation. **b** PDAC progresses from stage 1, a localized tumor, to stage 4, with extensive metastasis to the liver and other organs. At stage 4, PDAC is inoperable. Stage 2 is characterized by spread to nearby lymph nodes (borderline resectable), whereas stage 3 is characterized by local invasion to lymph nodes, blood vessels and nerves (mostly not resectable). Each stage can be further subdivided by including tumor size, the number of lymph nodes involved and metastatic spread. The size and number of pancreatic nerves increase as PDAC progresses from stage 1 to stage 4. Created in BioRender.com

In addition to the paracrine and autocrine interactions between tumor cells and local nerves, it is important to consider the effect(s) of hyperactivation of the autonomic nervous system (ANS) on cancer development. Stress, a highly conserved response in evolution to a perceived threat, can be acute (i.e., short-lived), and beneficial or chronic (i.e., occurring over an extended period) and detrimental, leading to pathophysiology, including cancer.^315^ As depicted in Fig. 4a, the proposed biological mechanisms linking chronic stress and cancer development include alterations in the hypothalamus‒pituitary‒adrenal (HPA) axis, which controls glucocorticoid release,^316^ and the sympathetic nervous system (SNS), which regulates catecholamine levels.^317,318^ The SNS, which innervates virtually every major organ system, reacts to stress perceived by the brain by releasing the catecholamine neurotransmitter norepinephrine (aka noradrenaline) from sympathetic nerve endings throughout the body and epinephrine (aka adrenaline) from the adrenal medulla into the bloodstream.^315,319^ In turn, these catecholamines elicit biological responses by acting through α-adrenergic receptors and β-adrenergic receptors, including ADRB1, ADRB2 and ADRB3. Accumulating evidence suggests that chronic stress accelerates the progression of a variety of malignancies through the activation of β-adrenergic signaling pathways,^318,320–325^ including PDAC,^307,326^ and that psychological stress is associated with cancer mortality.^327^ The progression of PDAC through different stages (1–4) of the disease is schematically depicted in Fig. 4b.

Preclinical studies reported that stress increases the growth of human and murine pancreatic tumor xenografts.^328,329^ For example, a combination of acoustic and restraint daily stresses enhanced the growth of pancreatic tumor xenografts and allografts in mice.^328^ The increase in pancreatic cancer xenograft growth induced by stress was attenuated by the administration of β-adrenergic receptor antagonists.^307,330^ In genetically modified mice expressing mutated *Kras* in pancreatic cells, repeated daily restraint stress or the administration of β-adrenergic receptor agonists enhanced PDAC development, primarily through catecholamine signaling.^312^ Epidemiological studies support the hypothesis that cardiovascular patients treated with nonselective β-adrenergic receptor antagonists have a reduced incidence of cancer^331^ and suggest that patients receiving β-blockers have a lower PDAC mortality rate than nonusers do,^326,332^ although outcomes depend on the type and dose of blocker and duration of treatment.^333^ The administration of β-blockers also increased cancer-specific survival in other cancers, including breast, ovarian, prostate and colorectal cancers.^334^ Furthermore, plasma adrenaline levels negatively correlate with overall survival in PDAC patients.^335^ Specifically, patients with low levels of circulating adrenaline had a median survival of 16.6 months, whereas patients with high adrenaline levels had a median survival of 9.6 months.^335^ Major stressful life events have also been suggested to function as risk factors in PDAC development.^336^ In addition to the SNS, glucocorticoids released from the adrenal gland during chronic stress increase neutrophil-mediated pancreatic cancer metastasis to the spleen, thus contributing to the aggressiveness of the disease.^337^

In contrast to the growing evidence supporting a critical role of the SNS in cancer development and progression, the outcomes of studies attempting to define the contribution of the parasympathetic (cholinergic) system to PDAC development are conflicting, at least in part because of the participation of different receptor systems, namely, nicotinic acetylcholine receptors (nAChRs) and muscarinic acetylcholine receptors (mAChRs), in different pancreatic cell types. In one study, cholinergic signaling via nAChRs, which are ligand-gated ion channels, promoted an immune-suppressive microenvironment in the pancreas that favored tumor growth in vivo, and bilateral vagotomy improved survival.^338^ In contrast, a previous study reported that cholinergic signaling via muscarinic acetylcholine receptor M1 (mAChR1), which is a GPCR, suppressed tumorigenesis through the inhibition of ERK and AKT signaling in PDAC cells.^339^ The elucidation of the role of cholinergic signaling via nicotinic and muscarinic receptors in PDAC and immune cells as well as the effect of subdiaphragmatic bilateral vagotomy on PDAC development requires further experimental work. In conclusion, substantial evidence suggests that chronic stress has a highly significant effect on the interaction between nerves and cancer cells, primarily through activation of the SNS, driving the progression of stage 1 (localized tumor) to stage 4 (metastatic PDAC) PDAC and depressing antitumor immunity,^307,318^ as summarized in Fig. 4.

Social isolation in humans is a stressor of high clinical significance. Indeed, loneliness and social isolation, which are prevalent in humans, increase the risk for premature death by 26% and 29%, respectively.^340,341^ Epidemiological data in humans strongly support the concept that a lack of social connections is associated with an increased risk of cancer and increased cancer mortality,^342–345^ and differences between men and women may exist. While men are generally more socially isolated than women are, females usually exhibit greater behavioral and neural responses to social isolation.^346,347^ The positive associations of social isolation and loneliness with cancer risk warrant more mechanistic studies in animal models to test whether social isolation increases cancer risk and growth. As discussed above, obesity induced by a high-fat diet is recognized to promote PDAC development, but despite its importance, the potential interaction between chronic stress and diet-induced obesity in the promotion of PDAC remains highly underexplored.

### Cooperation of chronic stress and obesity in promoting PDAC

Recent studies revealed a complex interplay between chronic stress, diet-induced obesity, and insulin resistance.^348–353^ Social isolation stress (SIS) induces obesity in wild-type mice^354^ and humans^355^ and leads to insulin resistance and compensatory hyperinsulinemia.^356^ In turn, diet-induced obesity, particularly visceral obesity, leads to SNS hyperactivation and an increase in catecholamine levels,^357,358^ which are thought to play important roles in the pathogenesis of obesity-associated cardiovascular and metabolic disorders and exacerbate stress-induced hyperphagia.^359^ There are multiple plausible ways by which obesity can lead to SNS activation, including hyperinsulinemia and hyperleptinemia.^360–362^ Recently, overnutrition has been shown to lead to insulin resistance and metabolic disturbances via increased SNS activity and the production of catecholamines, stimulating lipolysis.^363^

An elegant study in wild-type mice demonstrated that chronic stress combined with a high-fat diet results in insulin derepression of NPY neurons in the central amygdala, thereby causing exaggerated obesity and hyperinsulinemia.^352^ Specifically, mice subjected to chronic stress combined with a high-fat diet developed aggravated obesity due to an increase in caloric intake and a reduction in energy expenditure. This study also demonstrated a key role of insulin receptor signaling in controlling the activity of NPY neurons.^352^ These findings are highly important given that preclinical and epidemiological lines of evidence indicate that diet-induced obesity or chronic stress promotes cancer development and dissemination. However, despite its importance, the impact of stress and DIO on advanced PanIN formation and PDAC promotion in mice expressing mutated *Kras* in pancreatic cells has received little attention.

These considerations prompted us to explore the impact of social isolation, a recognized stressor for mice,^364^ on PDAC development in a KC mouse model in which a control diet or a high-fat diet was used to induce obesity. Socially isolated animals have been shown to develop behavioral, neurochemical, and hormonal changes, including increased catecholamine levels, increased systemic and local inflammation, and immune suppression. Social isolation (single housing) increased the proportion of PanIN-3 lesions in KC mice receiving a high-fat caloric diet (HFCD).^313^ These recent results imply that chronic isolation stress cooperates with diet-induced obesity to promote high-grade PanIN lesions in mice expressing a mutant Kras in pancreatic cells.^313^ Interestingly, *Adrb1* and *Adrb2* gene expression is increased in the pancreatic cells of KC mice fed a HFCD and subjected to social isolation stress (compared with those fed a HFCD), suggesting increased β-adrenergic receptor signaling in the pancreas under conditions of chronic stress and a HFCD.^313^ Given the cooperation between social isolation stress and diet-induced obesity in promoting PanIN-3 lesions in KC mice, the identification of the molecular node(s) involved in the convergence and integration of obesity mediators and stress neurotransmitters is emerging as an area of great interest.

## CREB phosphorylation in the action of stress neurotransmitters and obesity mediators in PDAC cells

In addition to the signaling pathways discussed in the preceding sections, recent studies identified the phosphorylation of the nuclear transcription factor cyclic adenosine monophosphate (cAMP) response element-binding protein (CREB) at Ser-133 as a stimulus of acinar-ductal metaplasia, PDAC development and metastasis.^89,365–368^ Phosphorylated CREB binds to cyclic AMP-responsive elements (CREs) in gene promoters^369^ and recruits the transcription coactivators CREB binding protein (CBP) and p300,^369–371^ thereby promoting the transcription of select genes in different cell types. In addition, phosphorylated CREB binds to mutant p53, thus inducing the expression of genes leading to PDAC metastasis.^89^ The expression of CREB is increased in a variety of neoplastic diseases, including pancreatic cancer.^372^ Activating transcription factor 1 (ATF1), a member of the CREB family, is also regulated by phosphorylation (on Ser-63) and plays an important role in cancer stem cell biology and as a pro-oncogenic factor in various cancers, including lung,^373^ colorectal^374^ and clear cell sarcoma.^375^ Given the similarity in the amino acid sequence surrounding CREB Ser-133 and ATF1 Ser-63, these transcription factors are likely to be phosphorylated by the same protein kinases.

Since the phosphorylation of CREB and ATF1 is necessary for their activation as transcription factors, defining the upstream protein kinases that mediate CREB and ATF1 phosphorylation in pancreatic cancer cells is important. Mutated KRAS has been proposed to induce CREB phosphorylation at Ser133 through ERK/RSK in PDAC cells.^89^ However, recent findings revealed very low levels of CREB phosphorylation in human PDAC cells harboring oncogenic *KRAS* that were incubated in serum-deprived medium.^313^ Since mutated KRAS expression is not sufficient to stimulate CREB phosphorylation, an additional stimulus (or stimuli) is likely necessary. In what follows, we discuss that stress neurotransmitters and obesity mediators stimulate protein kinases that mediate CREB phosphorylation in PDAC cells.

CREB and ATF1 can be phosphorylated at the critical Ser-133 and Ser-63 residues, respectively, by cAMP/PKA. Although cAMP was initially regarded as a negative regulator of cell proliferation, subsequent studies demonstrated that this second messenger stimulates the proliferation of a variety of cell types, including fibroblasts, thyroid epithelial cells, pancreatic cells, and macrophages.^376–378^ cAMP acts via three effectors, including PKA, GTPase exchange proteins directly activated by cAMP (EPACs), and cAMP-gated ion channels. PKA, a tetramer comprising two catalytic subunits (C) and two regulatory subunits (R), is the best characterized cAMP effector. The binding of cAMP to the R subunits liberates the functional C subunits, which are then able to phosphorylate nearby substrates in the cell.^379^ The R subunits modulate PKA signaling in different cellular locations through binding interactions with a class of scaffold proteins called A-kinase anchoring proteins (AKAPs).^380^ AKAPs constitute a large protein family that plays an important role in cAMP signaling via PKA.^381,382^

In the context of PDAC, either norepinephrine or the β adrenergic receptor agonist isoproterenol were found to increase CREB phosphorylation at Ser-133.^313^ Consequently, it is conceivable that neurotransmitters released by SNS fibers surrounding pancreatic cancer cells stimulate CREB/ATF1 phosphorylation via PKA, thus providing a molecular mechanism of action of neural inputs in pancreatic cells harboring oncogenic *KRAS*. Neurotensin, a neuropeptide implicated in stress,^383^ obesity,^277,279^ PDAC cell signaling^197^ and xenograft growth^278^ also elicits a rapid increase in CREB phosphorylation at Ser133 in PDAC cells.^313^ It is important to identify the protein kinase that mediates CREB phosphorylation in response to obesogenic neurotensin, given that substantial evidence indicates that CREB can also be phosphorylated at Ser-133 not only by PKA but also by several basophilic protein kinases.^384^

**Fig. 5:**
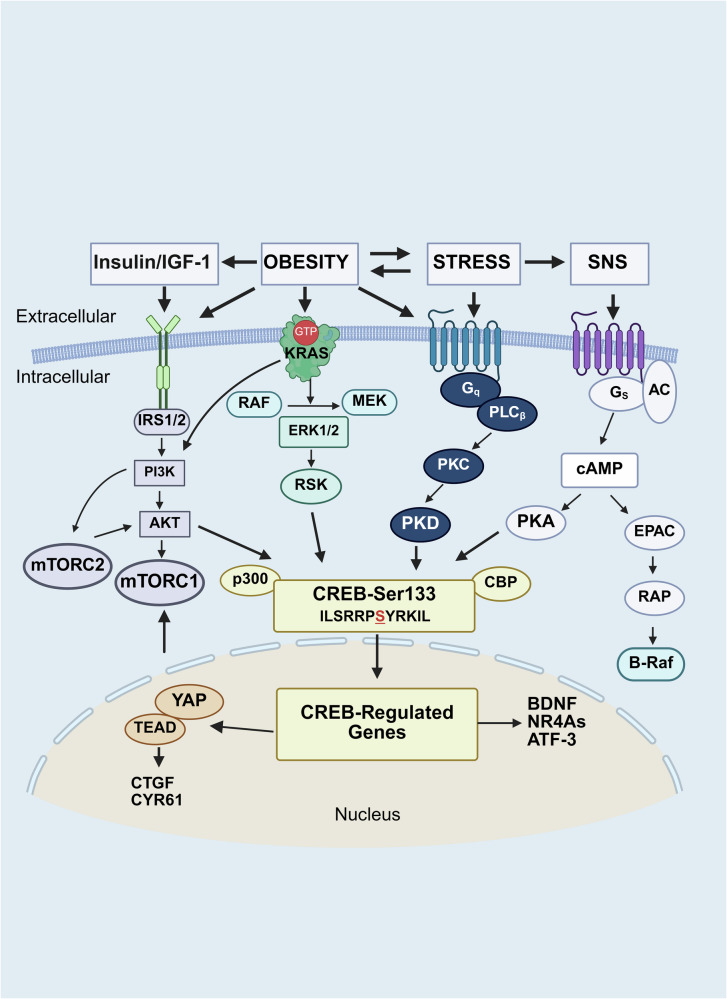
Obesity and stress induce molecular pathways that converge to promote activation of the transcription factor CREB in pancreatic cancer cells. Obesity mediators (e.g., neurotensin) and sympathetic nervous system (SNS) neurotransmitters (e.g., noradrenaline) bind to GPCRs, leading to the activation of protein kinases, including PKA and PKD, which phosphorylate CREB at Ser133. The amino acid sequence surrounding Ser-133 is a consensus for PKA and PKD, thus explaining the convergence of these protein kinases on CREB. Additional details can be found in the text. Created in BioRender.com

The amino acids neighboring CREB Ser-133 and ATF1 Ser-63 conform to a consensus protein kinase D (PKD) family phosphorylation site^385^ prompted us to speculate that kinases of the PKD family^386^ phosphorylate CREB and ATF1 in PDAC cells. PKDs, which are characterized by structural and regulatory properties,^385,386^ have emerged as critical nodes in cell signaling.^385,386^ PKDs are rapidly activated by neurotensin,^197,284^ whose G protein-coupled receptor (NTSR1) is overexpressed in highly malignant pancreatic cancer sublines.^278^ PKD has been implicated in the control of PDAC cell proliferation, invasion and acinar-to-ductal metaplasia,^387^ and a selective PKD family inhibitor markedly decreased PDAC cell growth in vitro and in vivo.^388^ Furthermore, single nucleotide polymorphisms in *PRKD1* (which encodes PKD1) have been associated with an increased risk for obesity,^389^ and PKD1 has been linked to the regulation of insulin secretion from the β cells of the pancreas.^390^ In this manner, PKD1 provides a potential molecular connection between obesity, insulin secretion and PDAC development. At the organismal level, PKD functions as a novel nutrient sensor^391^ and a major contributor to pathologies associated with obesity.^392^ Recent evidence indicates that the PKD family catalyzes CREB phosphorylation in PDAC cells stimulated by neurotensin.^313^ Consequently, obesity mediators and stress neurotransmitters stimulate protein kinase signaling pathways, including PKD and PKA, which converge on CREB/ATF1 phosphorylation in pancreatic cancer cells, as depicted schematically in Fig. 5. In line with this proposition, KC mice subjected to diet-induced obesity or social isolation stress presented a significant increase in PanIN cells positive for phosphorylated CREB. KC mice subjected to both diet-induced obesity and isolation stress presented further enhancement of phosphorylated CREB in PanINs.^313^

Notably, in some cell types, phosphorylation might not be sufficient to promote CREB-dependent transcription, suggesting the existence of additional posttranslational modifications regulating CREB activity, including acetylation, sumoylation, glycosylation and ubiquitination.^393^ It is not known whether any of these putative CREB modifications occur at any stage of PDAC development in response to obesity, stress or a combination of obesity and stress.

### Signaling downstream of CREB

CREB has been implicated in the regulation of the expression of multiple genes involved in the control of cell proliferation, survival, migration, differentiation, metastasis, metabolism and extracellular matrix production.^89,308,366,372^ However, the gene networks activated downstream of CREB are tumor type-specific and remain incompletely understood.^394^ Here, we focus on plausible downstream targets of CREB that regulate subsequent biological processes of major interest in the context of PDAC, including *NR4As*, *ATF3*, *YAP* and *FOXA1*.

The genes of the NR4A subfamily,^395,396^ which comprises NR4A1 (Nur-77), NR4A2 (Nurr-1), and NR4A3 (Nor-1), are structurally related orphan transcription factors that are rapidly upregulated via CREB in neuronal and solid tumor-derived cells.^395,397^ Subsequent studies implicated NR4A transcription factors in the control of cellular proliferation and metabolism in a tissue-dependent manner.^396^ NR4A1 is overexpressed in PDAC tissues, and its inactivation in PDAC cell lines decreases the proliferation and expression of prosurvival genes, including survivin.^398^

In neuronal cells, the CREB family of transcription factors are the main regulators of brain-derived neurotrophic factor (*BDNF*) gene expression,^399^ and emerging evidence suggests that NR4A2 mediates BDNF expression in these cells.^400^ It is conceivable that a CREB/NR4A2/BDNF signaling pathway activated via PKA or PKD in PDAC cells mediates a positive feedback loop driving pancreatic innervation in the tumor microenvironment and thereby promoting PDAC growth. Furthermore, the activation of the CREB/NR4A2/BDNF axis by obesogenic hormones could provide a molecular mechanism by which diet-induced obesity positively affects the sympathetic innervation of the pancreas, thus amplifying the effects of chronic stress via the SNS.

CREB has also been implicated in regulating the expression of activating transcription factor 3 (ATF3),^401^ another member of the CREB/ATF family of transcription factors. Recent evidence indicates that ATF3 is a tumor-promoting transcription factor in PDAC cells.^402–405^ Accordingly, ATF3 plays a role in the progression and maintenance of high-grade PanIN lesions in the pancreas,^402^ and other studies have implicated ATF3 in promoting perineural invasion^403^ and PDAC chemoresistance.^404^ Furthermore, ATF3 increases serine biosynthesis and tumor growth under dietary serine restriction.^405^ Given its importance as a PDAC tumor-promoting transcription factor, the regulation of the CREB/ATF3 axis in response to obesogenic mediators and stress neurotransmitters in these cells warrants further experimental work.

Another putative downstream target of CREB is YAP, which, as discussed in the previous sections, activates transcription with the TEAD1-TEAD4 DNA-binding proteins.^406^ Interestingly, studies using different cell types have shown that CREB promotes YAP mRNA expression^407–410^ and reciprocally that YAP forms a physical complex with CREB that increases the stability of phosphorylated CREB.^407,411^ These findings suggest that the interaction between CREB and YAP results in a positive feedback loop that further amplifies CREB-mediated PDAC development in response to an obesogenic diet and chronic stress.

Other downstream targets of CREB in the later stages of PDAC include members of the FOXA family of pioneer transcription factors that regulate cell growth, differentiation, and embryogenesis.^412^ As mentioned above, mutant p53 and CREB phosphorylated at Ser133 form a physical complex in PDAC cells that upregulates FOXA1 (Forkhead Box A1), in turn activating its transcriptional network to drive PDAC metastasis.^89^ Therefore, CREB plays a critical role in mediating PDAC development in response to stress and obesity and, subsequently, in the later stages of PDAC, synergizes with p53 to stimulate metastatic dissemination.

## Approaches for the treatment of PDAC

PDAC is an aggressive disease with no effective treatment and is projected to become the second cause of cancer fatalities during the next decade. The current standard treatment is chemotherapy with either FOLFIRINOX (a combination of 5-fluorouracil, oxaliplatin, irinotecan, and leucovorin) or gemcitabine and nab-paclitaxel.^413,414^ Compared with gemcitabine, FOLFIRINOX has better antitumor effects but is associated with higher rates of adverse events; thus, FOLFIRINOX is appropriate for patients with better performance status. The use of adjuvant and neoadjuvant chemotherapy in patients with PDAC has been comprehensively reviewed.^1^ Considerable efforts have focused on the tumor microenvironment for therapy, but targeting the desmoplastic stroma or immunosuppressive pathways has not been effective thus far, although some targets, including focal adhesion kinase (FAK), are being pursued.^415^ Disappointingly, multiple clinical trials to identify better treatments for PDAC have met with very limited success.

Here, we focus on new developments in targeted therapeutic approaches. As discussed in the preceding sections, a better understanding of the mechanisms leading to PDAC is highly important for identifying novel targets, prognostic biomarkers, and preventive strategies. The development of patient-derived organoids for in vitro assessment of candidate inhibitors is another step toward precision medicine in advanced pancreatic cancer.^416^ However, these models need to include the tumor microenvironment, which is increasingly considered a major player in the pathogenesis of the disease and a potential target in PDAC treatment.^415^

### Targeting the PI3K/AKT/mTOR and RAF/MEK/ERK pathways

The PI3K/Akt/mTORC1 module, depicted in Fig. 2, is active in human cancers, including PDAC, and consequently has emerged as a plausible therapeutic target. The combination of capecitabine and everolimus (RAD001, an allosteric inhibitor of mTORC1 of the rapamycin family) was modestly effective in patients with advanced pancreatic cancer.^417^ A list of completed and ongoing trials using mTORC1 inhibitors is provided in ref.^418^ Studies targeting upstream components of the mTORC1 pathway obtained comparable disappointing results. For example, a clinical trial investigating the antitumor activity of buparlisib (a PI3K inhibitor) and trametinib (a MEK inhibitor) revealed minimal effects in PDAC patients.^419^ In additional studies, administration of the AKT inhibitor MK2206 did not produce clinical benefits in advanced PDAC.^420^

As mentioned previously, the RAF/MEK/ERK cascade is a major growth-promoting pathway in PDAC and other cancers, and suppression of this pathway by targeted inhibitors is considered an important approach.^421^ However, targeting of MEK with trametinib, pimasertib or ERK with ulixertinib^422^ has been discouraging in clinical trials (reviewed in^423^). Combinations of trametinib with everolimus showed modest clinical efficacy but resulted in frequent treatment-related toxicity,^424^ whereas trametinib combined with the CDK4/6 inhibitor ribociclib was ineffective.^425^

**Fig. 6:**
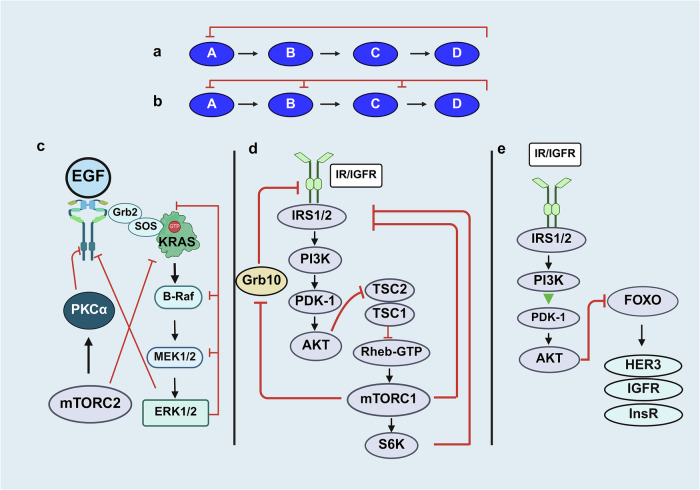
Growth-promoting pathways mediate negative feedback loops via phosphorylation of one or more upstream components of the pathway. **a** Example of a simple negative feedback loop in an ideal pathway in which a downstream component inhibits an upstream step, thereby turning off the pathway. **b** Example of more complex negative feedback regulation in an ideal pathway in which a downstream component inhibits multiple upstream steps, thereby turning off the pathway. **c** ERK mediates feedback inhibition of multiple upstream components, including MEK, RAF, SOS and EGFR. The inhibition of EGFR is mediated by phosphorylation at Thr-669. PKCα also mediates negative feedback via EGFR phosphorylation at Thr654. **d** mTORC1/S6K phosphorylates upstream regulators that participate in feedback loops that inhibit signaling through insulin/IGF receptors and other tyrosine kinase receptors. **e** Inhibition of AKT derepresses FOXO-mediated expression of tyrosine kinase receptors, including HER3, IGFR and InsR, thereby leading to drug resistance. More details concerning the importance of unleashing negative feedback loops in driving drug resistance can be found in the text. Created in BioRender.com

There are several reasons for these negative results, including limited information about biomarkers, a patient population with very advanced disease (i.e., stage 3–4), and the identification of challenging toxicities in human studies. The selection and stratification of PDAC patients for adjuvant treatment on the basis of biomarker-guided trials might be critical to improve outcomes.^426,427^ Furthermore, many critical signaling pathways not only induce downstream events that stimulate cell proliferation and migration but also mediate negative feedback via the phosphorylation of one or more upstream regulators, as depicted in Fig. 6a, b. For example, ERK mediates potent feedback inhibition of the upstream components of the pathway, including MEK,^428^ RAF^429,430^ and EGFR^431^ (Fig. 6c). Consequently, the inhibition of ERK releases these feedback inhibitory loops, leading to drug resistance.

Similarly, the mTORC1/S6K axis not only stimulates cellular growth but also phosphorylates upstream regulators, including the adaptor protein GRB10, which participate in feedback loops that inhibit PI3K/AKT through insulin/IGF receptors and other tyrosine kinase receptors in both normal and oncogene-transformed cells.^432^ Interruption of these feedback loops by inhibitors of mTORC1/S6K causes overactivation of upstream components of the pathway, including PI3K and AKT, which potentially oppose the antiproliferative effects of the inhibitors and lead to drug resistance (Fig. 6d). These findings provide an impetus to identify inhibitors that block both PI3K and mTOR, which are related kinases, but these new generation inhibitors revealed new feedback loops. For example, the dual PI3K/mTOR inhibitor BEZ235 inhibited mTOR without causing overactivation of Akt. However, BEZ235 and other dual PI3K/mTOR inhibitors (e.g., PKI-587 and GDC-0980) induced ERK activation, possibly by blocking a previously unknown feedback loop mediated by mTORC2^433^ (Fig. 6c). Furthermore, long-term inhibition of AKT derepresses FOXO-mediated expression of tyrosine kinase receptors, including HER3, IGFR and InsR,^434,435^ thereby leading to drug resistance (Fig. 6e). Although future trials may include inhibitors of PI3K/Akt/mTORC1 or RAF/MEK/ERK as part of low-dosage drug combinations to avoid challenging toxicities and release of negative feedback loops, the current attention is in the development of novel inhibitors that directly target KRAS and YAP/TEAD, as discussed below.

### Targeting RAS

Given the critical role of KRAS in PDAC, a major effort has been made to identify KRAS inhibitors. The last decade witnessed striking advances in identifying molecules that directly target RAS.^436,437^ The compounds sotorasib^438^ and adagrasib^439^ specifically bind covalently to KRAS^G12C^ and show favorable clinical activity in nonsmall lung cancer (NSCLC) patients. As indicated above, *KRAS* mutations in PDAC also occur at position G12,^44^ but with a G12D single amino acid substitution being the most prevalent.^45^ The cysteine mutation in G12C is found in only 1–2% of PDACs, prompting the search for additional RAS inhibitors to target this disease.

Building on the development of G12C inhibitors and the identification of a pocket in KRAS that was not identified in previous structural studies,^440^ new inhibitors have been described, including MRTX1133, which was identified as a noncovalent selective KRAS^G12D^ inhibitor that is active at nanomolar concentrations.^441–443^ However, drug resistance caused by feedback reactivation of upstream receptor tyrosine kinase (RTK) signaling through wild-type RAS significantly limits the activity of MRTX1133.^444^ For example, MRTX1133 increases the expression and phosphorylation of EGFR and HER2,^445^ and MRTX1133 acts synergistically with anti-EGFR drugs (e.g., erlotinib and lapatinib) in patient-derived organoids.^416^ Therefore, broad-spectrum multi-RAS inhibitors are likely to produce more durable antitumor responses than are selective KRAS^G12D^ inhibitors. In this context, new compounds attracting a great deal of interest include daraxonrasib (RMC-6236), a noncovalent inhibitor of the active, GTP-bound state (ON) of both mutant and wild-type variants of RAS isoforms.^446,447^ RMC-7977 is a compound related to RMC-6236 that has also been identified as a potent and orally active reversible inhibitor of the active GTP-bound state of both mutant and wild-type variants of KRAS, NRAS and HRAS.^448,449^ RMC-6236 and RMC-7977 bind with high affinity to the chaperone protein cyclophilin A and then form a complex with RAS proteins, thereby sterically impairing the interaction of RAS with its effectors.^447,449^ Preclinical characterization of these broad-spectrum RAS (ON) inhibitors has shown that these novel compounds inhibited the growth of an extensive panel of cells harboring RAS mutations and exhibited tumor-suppressive activity in mouse models of PDAC and other types of cancer, with minimal toxicity to normal cells from the skin or colon.^446–449^ The possibility of combining this class of KRAS inhibitors with immunotherapy has recently been proposed.^450^

RMC-6236 has entered clinical trials as a monotherapy and in combination with other therapies (ClinicalTrials.gov identifiers: NCT05379985; NCT06040541; NCT06040541). A new phase 3 multicenter trial (RASolute 302) is recruiting to compare RMC-6236 with standard care chemotherapies (NCT06625320). Reviews concerning the synthesis, pharmacological effects and ongoing clinical trials of RMC-7977 and RMC6236 have appeared recently.^451,452^ While the progress made in targeting RAS proteins is enormous, a major challenge for the use of KRAS inhibitors in the clinic is the development of resistance, primarily via the upregulation of YAP/TAZ activity, as discussed below. The long-term efficacy of KRAS inhibitors is most likely attained through combination therapy.^453^

### Targeting YAP/TEAD

As discussed above, YAP is a main pro-oncogenic factor in PDAC and plays a key role in circumventing KRAS function in the basal-like/squamous subtype of the disease, thereby mediating resistance to the KRAS inhibitor MRTX1133.^454^ Although targeting transcription factors is a challenging strategy, recent efforts have made major advances in targeting YAP/TEAD. Specifically, inhibitors that target TEAD proteins have been identified.^455^ Some of the compounds inhibit YAP/TEAD-regulated transcription by targeting a hydrophobic pocket in TEAD, which is necessary for complex formation with YAP and TAZ, whereas an additional class of TEAD inhibitors bind to an allosteric site on the surface of the TEADs and block their binding to YAP.^456–458^ For example, GNE-7883 prevents interactions between YAP/TAZ and TEAD through binding to the TEAD lipid pocket.^458^ Other compounds, including IAG933 and its analogs, act as selective disruptors of the YAP‒TEAD protein‒protein interaction and synergize with the KRAS^G12D^ inhibitor MRTX1133.^459^

Although broad-spectrum RAS inhibitors can prevent resistance via the activation of wild-type RAS proteins by RTK activation, relapse also occurs. For example, in a KPC mouse model, treatment with the pan-RAS inhibitor RMC-7977 markedly increased survival, but it was followed by relapse.^448^ Analysis of reverted tumors revealed *Myc* copy number gain as the predominant resistance mechanism, which was mediated by YAP/TAZ activation. Accordingly, treatment with the TEAD inhibitor IAG933^459^ overcame resistance to RMC-7977 in vitro.^448^

Although cotargeting RAS and YAP/TEAD is emerging as an attractive therapeutic approach that warrants further investigation, resistance to TEAD inhibitors via RAF/MEK/ERK pathway activation leading to FOSL1 also develops.^460^ In this case, inhibitors of the RAF/MEK/ERK pathway might be repurposed for overcoming resistance to TEAD inhibitors. Given the importance of YAP tyrosine phosphorylation for its nuclear localization (discussed above), an alternative approach to overcome resistance to RAS inhibitors is to inhibit YAP function by blocking SFKs with dasatinib.^248^

### Immunotherapy and mRNA vaccines

Cancer immunotherapeutic approaches, including anti-programmed cell death protein 1 (PD1) and anti-cytotoxic T-lymphocyte protein 4 (CTLA4), have not been effective in treating PDAC.^461^ For example, the results from a randomized phase II trial (NCT02879318) comparing gemcitabine and nab-paclitaxel with and without the immune checkpoint inhibitors durvalumab and tremelimumab in patients with metastatic PDAC revealed that combination immunotherapy did not improve survival.^462^ These negative results are most likely the consequence of the highly immunosuppressive microenvironment^33^ that characterizes PDAC.^32^ A major challenge is that all major cell types residing in the tumor microenvironment can repress T-cell responses, facilitating immune evasion.^33^ A number of clinical trials in which various signaling pathway inhibitors are combined with immunotherapy are underway (reviewed in ref. ^32^). Interestingly, clinical studies have demonstrated that a small subset of PDAC patients with high microsatellite instability benefit from immunotherapy,^463,464^ which highlights the importance of genetic testing of all patients with this disease and the importance of personalized medicine. A new approach targeting both T-cell subsets with antitumor activity and immunosuppressive myeloid cells yielded positive results in preclinical models, suggesting a possible way forward for immunotherapy in patients with PDAC.^465^

Recurrence of PDACs after resection is a feature of the disease that aggravates its prognosis. An initial phase I study evaluated a personalized mRNA vaccine for surgically removed pancreatic cancer.^466^ The vaccine elicited neoantigen-specific T cells in 8 of 16 patients with surgically resected PDAC, leading to T-cell expansion and delays in disease recurrence. A recent report provided long-term follow-up data from the initial phase I study.^467^ Immune responders continue to have prolonged recurrence-free survival and exhibit circulating long-lived CD8^+^ T-cell clones.^467^ Although the number of patients is small, this represents an exciting development. A major multicenter clinical trial comparing personalized mRNA vaccines combined with modified leucovorin, 5-fluorouracil, irinotecan, and oxaliplatin (mFOLFIRINOX) versus mFOLFIRINOX alone in participants with resected PDAC is now in progress (NCT05968326).

## Approaches for the prevention of PDAC

As discussed in the preceding sections, obesity, metabolic syndrome, and stress are associated with an increased risk of PDAC according to epidemiological and preclinical studies. Since these conditions function as tumor promoters, their effects are reversible, and consequently, interventions targeting patients with obesity, metabolic syndrome and stress are plausible strategies for the prevention of PDAC.

### Targeting stress: β adrenergic receptor antagonists

As examined previously, accumulating evidence supports the concept that pancreatic innervation plays a role in PDAC development and that chronic stress promotes PDAC and other cancer types through increasing the activity of the SNS. The catecholamines released by the nerve endings of the SNS bind to β adrenergic receptors expressed by pancreatic cancer cells and thereby modulate their behavior. Catecholamines also act on a variety of cells in the tumor microenvironment, including immune cells, thereby allowing cancer cells to elude immune surveillance.^307,318,468^ Given the critical role of β adrenergic receptors in mediating the impact of stress neurotransmitters, including norepinephrine and epinephrine, the repurposing of β-blockers for the prevention/interception of PDAC is conceivable. The recognition of the importance of neural inputs in the development of PDAC and other cancer types has opened new avenues for translational applications.^469^ Epidemiological studies suggest that patients receiving β-blockers have a lower PDAC mortality rate than nonusers do^332,470–472^; however, as noted above, the outcome is not uniform and depends on the type and dose of blocker and duration of treatment.^333^ In this context, the impact of third-generation β-blockers, including carvedilol, on the incidence of PDAC remains unknown.

Some small trials testing the effects of β-blockers in PDAC patients have been performed or are underway (e.g., NCT05451043; NCT03838029; DRKS00014054; and NCT04245644). Larger clinical trials are needed to define the benefits of β-blockers and other stress-reducing interventions on survival outcomes in patients with PDAC. In addition, the parasympathetic system was also targeted in a phase 1 study in which the muscarinic receptor agonist bethanechol was used to treat localized PDAC prior to surgery (NCT03572283). YAP/TEAD inhibitors combined with β-blockers offer new avenues to target the CREB/YAP axis in the context of PDAC promoted by DIO and chronic stress.

### Targeting obesity and T2DM: metformin

Metformin (1,1-dimethylbiguanide hydrochloride) is the most extensively recommended drug for the prevention and treatment of T2DM worldwide. Systemically, metformin decreases glycemia mainly through reduced hepatic gluconeogenesis, although the precise mechanism responsible for this decline in glucose production remains incompletely understood, and the precise relationship between the antidiabetic and anticancer effects of metformin remains unclear.

**Fig. 7:**
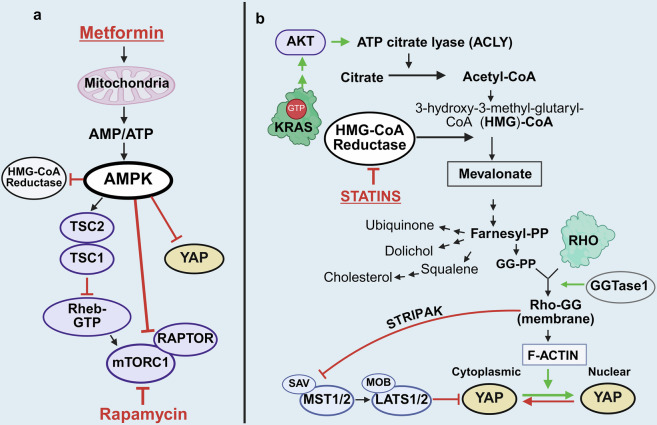
Mechanism of action of metformin (**a**) and statins (**b**). **a** Metformin inhibits the mitochondrial respiratory chain, thereby decreasing ATP synthesis, leading to AMP-activated protein kinase (AMPK), a heterotrimeric complex formed by the α, β and γ subunits. Active AMPK inhibits the function of pivotal growth-promoting pathways, including mTORC1 and YAP, as indicated. **b** Statins are specific inhibitors of 3-hydroxy-methylglutaryl (HMG) CoA reductase, the rate-limiting enzyme in the generation of mevalonate. The formation of mevalonate is the first step in the biosynthesis of isoprenoids, leading to farnesyl pyrophosphate (FPP), geranylgeranyl pyrophosphate (GG-PP) and cholesterol. The transfer of the geranylgeranyl (GG) moiety to a COOH-terminal cysteine of Rho GTPases is necessary for their membrane localization and function. Active Rho (i.e., Rho-GTP) plays a critical role in YAP/TAZ activation through actin remodeling and STRIPAK-mediated MST1 inactivation. Further details are provided in the text. Created in BioRender.com

As depicted schematically in Fig. 7a, metformin is thought to inhibit complex I of the mitochondrial respiratory chain, thereby decreasing ATP synthesis and leading to AMP–activated protein kinase (AMPK) activation.^473^ AMPK, a complex composed of α, β and γ subunits, is an energy sensor that is highly sensitive to increases in AMP/ATP and ADP/ATP ratios and thereby balances ATP consumption with ATP synthesis.^474^ Binding of AMP or ADP to the regulatory γ subunit induces a large conformational change,^475^ which facilitates the phosphorylation of the kinase activation loop in the catalytic α subunit by the tumor suppressor LKB-1/STK11 (liver kinase B1/serine–threonine kinase 11) at the highly conserved Thr172.^474^ Additional pathways of AMPK activation involving glucose sensing by aldolase in lysosomal membranes and axin have been identified.^476,477^

Previous studies using human PDAC cells demonstrated that treatment with metformin reduced the mitochondrial membrane potential and intracellular ATP levels, induced AMPK activation and concomitantly inhibited mTORC1, ERK and DNA synthesis.^478^ Knocking down AMPK prevents the inhibitory effect of low concentrations of metformin on mTORC1, ERK and DNA synthesis in PDAC cells.^478^ These studies emphasized that the effects of metformin on PDAC cells are steeply dose dependent: at low concentrations, metformin induced cellular effects via AMPK, whereas at high concentrations, metformin provoked cellular responses through AMPK-independent mechanisms. Notably, AMPK inhibits YAP/TAZ/TEAD function via direct phosphorylation^171,172,479^ and blocks mTORC1 activation (Fig. 7a). In preclinical models, the administration of metformin prevented the promotion of PDAC induced by DIO in KC mice, including the depletion of intact pancreatic acini, the formation of PanIN-3 lesions and increased PDAC incidence.^268,480^ These studies support the notion that metformin offers a potential approach for the prevention/interception of obesity-associated PDAC.

Epidemiologically, metformin administration has been associated with a reduced incidence, recurrence and mortality of pancreatic cancer in diabetic patients,^268,481^ although a beneficial effect was not observed in all studies,^482^ especially in patients with advanced PDAC. A recent study concluded that metformin users had a higher median overall survival (OS) of 29 vs 14 months and a better 5-year OS rate of 19% vs 5%.^483^ These studies suggest that the well-tolerated and inexpensive antidiabetic drug metformin impairs the promotion of PDAC either through direct inhibitory effects on pancreatic cells harboring an activating *Kras* mutation and/or indirectly through effects on the tumor microenvironment or extrapancreatic sites that sustain a chronic inflammatory state and amplify the progression of PDAC.^268^ However, other studies have shown no significant benefit. As in other cases, stratification and selection of patients are likely to be highly important. Given the emerging results indicating cooperation between chronic stress and obesity in promoting the formation of high-grade PanINs,^313^ novel combinatorial strategies, including propranolol and metformin, can be designed to prevent PDAC via the FDA-approved drugs discussed above.

### Targeting obesity and T2DM: GLP1 receptor agonists

The possibility of utilizing novel GLP-1 receptor agonists to reduce obesity-associated pancreatic cancer^484,485^ is an exciting prospect that deserves further experimental and clinical studies. Interest in this area has increased in recent reports suggesting that treatment of T2DM or obese patients with GLP-1 receptor agonists not only does not correlate with an increased incidence of PDAC^484^ but is also associated with a significantly decreased prevalence of pancreatic cancer compared with diabetic patients treated with insulin.^485^ A recent study also suggested a protective effect of GLP-1 receptor agonists^486^ but the possibility of adverse effects, including pancreatitis and PDAC, has been raised.^487^

### Targeting cardiometabolic disorders as risk factors for PDAC: statins

Several drugs are used to treat cardiometabolic disorders, including statins, which significantly affect signaling and proliferation in cultured PDAC cells and PDAC development in vivo. Many studies have shown that the mevalonate pathway is increased in epithelial cancers via mutant p53 and Akt/mTORC1.^488^ Statins are specific inhibitors of 3-hydroxy-methylglutaryl (HMG) CoA reductase, the enzyme that catalyzes the first step in the biosynthesis of isoprenoids, leading to farnesyl pyrophosphate (FPP), geranylgeranyl pyrophosphate (GG-PP) and cholesterol (Fig. 7b). The transfer of the GG moiety of GG-PP to the C-terminal cysteine of Rho GTPases is vital for their function in signal transduction. In turn, active Rho is crucial for YAP/TAZ activation through actin remodeling and activation of the striatin-interacting phosphatase and kinase (STRIPAK) complex, which inactivates the upstream kinases of the Hippo pathway.^198^ Rho activation in PDAC patients is associated with poor prognosis.^232^ In view of the mechanisms discussed in the preceding sections linking YAP/TEAD with PDAC, there is considerable interest in repurposing statins for the chemoprevention of pancreatic cancer. A high-throughput screen of compounds that interfered with the nuclear localization of YAP led to the identification of statins as potential YAP inhibitors,^489^ a notion substantiated in other studies.^490^ Additional studies have indicated that statins inhibit actin cytoskeleton remodeling, YAP/TEAD-regulated gene expression, proliferation, and colony formation in PDAC cells and attenuate acinar-ductal metaplasia.^491^

Observational studies imply that the use of statins is associated with a reduced risk and beneficial effects in patients with PDAC.^492–497^ A meta-analysis of 26 studies involving more than 3 million participants and 170,000 PDAC patients revealed a significant decrease in PDAC risk with statin administration.^495^ In contrast, statins do not have any effect on patients with advanced, nonresectable PDAC.^498^ Given that chronic stress and obesity cooperate in promoting the formation of high-grade PanINs, novel strategies to prevent PDAC via combinations of the FDA-approved drugs discussed above, including statins and propranolol, are possible. The available epidemiological and preclinical evidence warrants a comprehensive clinical evaluation of the chemopreventive role of statins in PDAC in at-risk populations.

## Concluding remarks

PDAC is a severe disease with no effective treatment and is anticipated to become the second foremost cause of cancer-related deaths in the US and Europe by 2030. A deep understanding of the molecular pathogenesis, signaling pathways and risk factors leading to PDAC is highly important for identifying novel targets, prognostic markers, preventive strategies, and signature markers for use in specific and individualized therapy. Active KRAS stimulates downstream signaling pathways that are essential for initiating PDAC but are not sufficient to promote the transition of preneoplastic PanIN lesions to overt PDAC. This process requires additional signaling inputs to drive pancreatic cell proliferation and PDAC promotion. Here, we highlight the complex interplay between KRAS signaling, the transcriptional coactivator YAP and SFKs in the development of PDAC, including the importance of these signaling proteins in metabolic reprogramming and in shaping the tumor microenvironment.

We also emphasize the importance of modifiable risk factors that function as tumor promoters of initiated pancreatic cells harboring *KRAS* mutations. In this context, diet-related metabolic disorders, including obesity, have been associated with increased risk and worse clinical outcomes for the development of PDAC in humans, and diet-induced obesity markedly accelerates the progression of low-grade PanINs to high-grade PanINs and PDAC in mice with mutated *Kras* in pancreatic cells. Accumulating evidence also indicates that neural signals regulate critical functions of cancer cells, including their proliferation and dissemination, and that chronic stress promotes PDAC through the sympathetic nervous system by acting via β-adrenergic receptors expressed by PDAC cells and influencing other cells in the tumor microenvironment. We also emphasize that obesogenic mediators and stress neurotransmitters stimulate protein kinase signaling pathways, including PKA and PKD, which converge on CREB/ATF1 phosphorylation in pancreatic cancer cells. We envisage CREB as a key node in a signaling network engaged by stress and obesity that promotes the progression of *KRAS*-initiated pancreatic cells.

Since emerging results indicate cooperation between stress and obesity in promoting PDAC, it is plausible to envisage novel combinatorial strategies to prevent PDAC, repositioning FDA-approved drugs that are extensively used to treat cardiovascular and metabolic disorders as potential chemopreventive interventions. It will also be important to examine the cancer-preventive effectiveness of combinations of FDA-approved agents at low concentrations of each class, including β-blockers, metformin, statins, and GLP-1 receptor agonists, and consider replacing first-generation β blockers with novel drugs (e.g., third-generation carvedilol). We also emphasize the identification of new molecules that directly target RAS proteins and YAP in PDAC as major areas of clinical research. Novel YAP/TEAD inhibitors provide a strategy to circumvent drug resistance caused by the release of negative feedback loops that fine-tune signaling downstream of RAS proteins. The development of novel approaches for the treatment and prevention of PDAC is a critical necessity that guarantees further mechanistic, preclinical, and clinical work.
